# Supplementary material for: Understanding transnational healthcare use in immigrant communities from a cultural systems perspective: a qualitative study of Dutch residents with a Turkish background
Source: BMJ Open. 2021 Sep 30;11(9):e051903. doi: 10.1136/bmjopen-2021-051903 (PMC8487186; doi:10.1136/bmjopen-2021-051903)
Supplement: Supplementary data [file bmjopen-2021-051903supp001.pdf]

Supplementary Material I. Interview approach

We used the biographic-narrative interview method (1). The first part of the interview was an open interview, the second part was structured around the episodes of health care use that the respondent had mentioned in the first part.

Part 1. Open query
"Lütfen bana hayatınızın hikayesini, sizin için sağlık, hastalık ve bakım ilgili önemli olan tüm olayları ve yasadıklarınızı bana anlatın. Başlamak istediğiniz yerde başlayın, ve istediniz kadar anlatın, vaktim çok, ben siz anlatınız an, sissiz kalıyorum, sadece sonra için birkaç not alacağım"

"Please tell me the story of your life, in the sense of all the events and experiences that have been important to you personally regarding health, sickness and seeking care. Start wherever you want to, and tell me as much as you want, I have a lot of time, and if I get confused, I'll make some notes for later"

Part 2. In-depth questions
Questions to further explore the respondent's experiences during the subsequent episodes of healthcare use. These questions were not pre-determined, but guided by (a) the topics raised by the respondent; and (b) the exact wording used by the respondent. In order to understand the respondent's experiences and explanatory model of illness, this part of the interview was structured around the three phases of the consultation (2, 3): (I) the presentation of illness sensations; (II) the transition of an illness into a disease; (III) presenting a treatment regime.

1. Wengraf T. Qualitative research interviewing: Biographic narrative and semistructured methods. Thousand Oaks, CA: Sage; 2001.
2. Kleinman A. Concepts and a model for the comparison of medical systems as cultural systems. Social Science & Medicine Part B: Medical Anthropology. 1978;12:85-93.
3. Hay MC. Reading Sensations: Understanding the Process of Distinguishing `Fine' from `Sick'. Transcultural Psychiatry. 2008;45(2):198-229.
