# Supplementary material for: Understanding transnational healthcare use in immigrant communities from a cultural systems perspective: a qualitative study of Dutch residents with a Turkish background
Source: BMJ Open. 2021 Sep 30;11(9):e051903. doi: 10.1136/bmjopen-2021-051903 (PMC8487186; doi:10.1136/bmjopen-2021-051903)
Supplement: Supplementary data [file bmjopen-2021-051903supp002.pdf]

Supplementary Material II. Codes used in the Qualitative Analysis

Steps of consultation (negotiation at each phase)	
	Illness presentation and persuading
	illness to disease translation
	Acceptable treatment regimen
Sensation to symptom	
	Vulnerability
	Urgency
	Disability/death (seriousness)
Core adaptive tasks of healthcare systems	
	Dealing with illness
	Healthcare strategies
	Consultation
	Healing activities
	Health influencing behaviours
	Dealing with healthcare outcomes
Explanatory model	
	Patient
	Provider
	Differences/troubles
	Agreement
Endpoint	
	Conflicting
	Unacceptable
	Acceptable
	Test result
	Diagnosis
	It's nothing serious
Turkish Healthcare	
	Direct access to specialist care
	Ruling in versus ruling out
	Interventionalist
	Value for money
	"Broad" & fast diagnostics
Dutch Healthcare	
	Specialist referral
	Ruling out versus ruling in
	Non-invertentionalist
	Stepwise diagnosis
	Stepwise treatment
Life World Intern	
	Turkish examples
	Dutch examples
	Cultural assumptions
Life World Extern	

Dutch setting experience
Turkish setting experience
Cultural context
Being an experiment
Self-reliance
Finances
Language
Delay
Shopping (behaviour)
Uncertainty avoidance
Transnationalism
Access without barriers
Why? Expressing lack of understanding
Metaphor
