# Supplementary material for: Understanding transnational healthcare use in immigrant communities from a cultural systems perspective: a qualitative study of Dutch residents with a Turkish background
Source: BMJ Open. 2021 Sep 30;11(9):e051903. doi: 10.1136/bmjopen-2021-051903 (PMC8487186; doi:10.1136/bmjopen-2021-051903)
Supplement: Author's manuscript [file bmjopen-2021-051903.draft_revisions.pdf]

BMJ Open

BMJ Open is committed to open peer review. As part of this commitment we make the peer review history of every article we publish publicly available.

When an article is published we post the peer reviewers' comments and the authors' responses online. We also post the versions of the paper that were used during peer review. These are the versions that the peer review comments apply to.

The versions of the paper that follow are the versions that were submitted during the peer review process. They are not the versions of record or the final published versions. They should not be cited or distributed as the published version of this manuscript.

BMJ Open is an open access journal and the full, final, typeset and author-corrected version of record of the manuscript is available on our site with no access controls, subscription charges or pay-per-view fees (http://bmjopen.bmj.com).

If you have any questions on BMJ Open's open peer review process please email info.bmjopen@bmj.com

Understanding transnational healthcare use in immigrant communities from a cultural systems perspective: A qualitative study of Dutch residents with a Turkish background

Journal:	*BMJ Open*
Manuscript ID	bmjopen-2021-051903
Article Type:	Original research
Date Submitted by the Author:	01-Apr-2021

Complete List of Authors:	Şekercan, Aydin; Amsterdam UMC Locatie AMC, Public and Occupation Health<br>Harting, Janneke; Amsterdam UMC Locatie AMC,<br>Peters, Ron; Amsterdam UMC - Locatie AMC, Cardiology<br>Stronks, Karien; Amsterdam UMC Locatie AMC, Public and Occupational Health

Keywords:	PRIMARY CARE, PUBLIC HEALTH, QUALITATIVE RESEARCH

SCHOLARONE™
Manuscripts

I, the Submitting Author has the right to grant and does grant on behalf of all authors of the Work (as defined in the below author licence), an exclusive licence and/or a non-exclusive licence for contributions from authors who are: i) UK Crown employees; ii) where BMJ has agreed a CC-BY licence shall apply, and/or iii) in accordance with the terms applicable for US Federal Government officers or employees acting as part of their official duties; on a worldwide, perpetual, irrevocable, royalty-free basis to BMJ Publishing Group Ltd ("BMJ") its licensees and where the relevant Journal is co-owned by BMJ to the co-owners of the Journal, to publish the Work in this journal and any other BMJ products and to exploit all rights, as set out in our licence.

The Submitting Author accepts and understands that any supply made under these terms is made by BMJ to the Submitting Author unless you are acting as an employee on behalf of your employer or a postgraduate student of an affiliated institution which is paying any applicable article publishing charge ("APC") for Open Access articles. Where the Submitting Author wishes to make the Work available on an Open Access basis (and intends to pay the relevant APC), the terms of reuse of such Open Access shall be governed by a Creative Commons licence – details of these licences and which Creative Commons licence will apply to this Work are set out in our licence referred to above.

Other than as permitted in any relevant BMJ Author's Self Archiving Policies, I confirm this Work has not been accepted for publication elsewhere, is not being considered for publication elsewhere and does not duplicate material already published. I confirm all authors consent to publication of this Work and authorise the granting of this licence.

For peer review only

TITLE

Understanding transnational healthcare use in immigrant communities from a cultural systems perspective: A qualitative study of Dutch residents with a Turkish background

AUTHORS

Aydın Şekercan[1,2], Janneke Harting[1], Ron J. Peters[3], Karien Stronks[1]

AFFILIATIONS

1 Amsterdam UMC, University of Amsterdam, Department of Public and Occupational Health, Amsterdam Public Health research institute, Amsterdam, the Netherlands

2 Dutch hospital "Onze Lieve Vrouwe Gasthuis", location East, Department of General Surgery, Amsterdam, the Netherlands

3 Amsterdam UMC, University of Amsterdam, Department of Cardiology, Amsterdam, the Netherlands

WORD COUNT

Main text: 3.995

CORRESPONDING AUTHOR

Janneke Harting

Amsterdam UMC, Department of Public and Occupational Health

Meibergdreef 15, 1105 AZ Amsterdam, the Netherlands

j.harting@amsterdamumc.nl

+31 20 566 50 49

For peer review only - http://bmjopen.bmj.com/site/about/guidelines.xhtml

ABSTRACT

Objectives

Transnational utilisation of healthcare by people with an immigrant background carries risks, including medicalisation and adverse iatrogenic outcomes. We investigated the drivers behind such transnational healthcare use from a cultural perspective on health systems.

Design

Qualitative interview study (2018).

Setting

Two primary care practices in Amsterdam, the Netherlands.

Participants

Thirteen Dutch patients of Turkish background, who had obtained healthcare in Turkey, and who in general visited the primary care practice more than once a month.

Results

We found that: (A) cross-border healthcare use was fostered by cultural mismatches between expected and provided services and by differing explanatory models of illness upheld by patients and Dutch providers; (B) both transnationalism in patients and entitlements to insurance reimbursement facilitated the use of Turkish health services to bypass perceived barriers in the Dutch system; (C) cultural mismatches were reinforced during GP consultations after the patients' return to the Netherlands, thereby inducing further service use abroad.

Conclusions

Although cultural system influences are difficult to bridge, measures to reduce the unwelcome consequences of transnational healthcare use may include (A) strengthening the provision of culturally sensitive care in the country of residence and (B) restricting the

For peer review only - http://bmjopen.bmj.com/site/about/guidelines.xhtml

reimbursement of care in the country of origin while maintaining the option to obtain care abroad.

KEY TERMS

migrants, cultural system, transnationalism, cross-border care, transnational healthcare

ARTICLE SUMMARY

Strengths and limitations of the study

- A first strength is our innovative approach to explore the drivers behind transnational healthcare use from a cultural systems perspective
- This cultural systems perspective helped us to understand how transnational healthcare use may be fostered by an interaction of factors in both clinical practice, healthcare system and national culture
- A second strength is the ethnic-concordant interviewer, familiar with the respondents' language and cultural expressions, and the biographic-narrative interpretive interview method, together leading to in-depth and meaningful information
- An important limitation is the rather small number of participants in the study, sampled from two primary care practices in one transnational healthcare context, which limits the generalizability of our finding to other populations and contexts

For peer review only

BACKGROUND

Across the globe, the transnational ties of former migrants and their families are enabling them to retain and pursue private, cultural and economic interests in their countries of origin, including the domain of healthcare. This phenomenon is known as transnationalism (1, 2). People with an immigrant background tend to utilise similar healthcare services in both their country of origin and their country of residence (1, 3). High transnational healthcare use may increase the risk of medicalisation, as different approaches in the assessment of illnesses may cause more ailments to be labelled as diseases. It also increases the risk of iatrogenic complications resulting from additional diagnoses and treatments (4). Upon the patients' return to their country of residence, they may be subject to repetitions of diagnostics, drug interactions in treatments and inadequate treatment aftercare, due to a lack of cross-border medical information transfers (5). Little evidence is available on the drivers that underlie frequent transnational healthcare use. Such information could be helpful in curtailing its adverse effects.

People of Turkish ethnic origin who are resident in the Netherlands are a relevant group for further study of this phenomenon. In this largest ethnic minority group in the Netherlands, transnational health service use is highly prevalent. Dutch residents with Turkish backgrounds are more likely to utilise health services in the country of origin than other migration groups – 46%, as compared to 18% of residents with Moroccan backgrounds (6). This happens even though apparently equivalent facilities are available in the Netherlands (7). Moreover, those who frequently use health services in Turkey also tend to be frequent healthcare users in the Netherlands (often in the top 10% of frequent users). Their overall rates of utilisation are also high in comparison with their co-ethnics who do not obtain healthcare in Turkey (6, 8). As 80% of the Dutch residents of Turkish background

For peer review only - http://bmjopen.bmj.com/site/about/guidelines.xhtml

maintain ties with the Turkish culture, they can readily compare, utilise and evaluate various features of health services in both countries (2, 5). There is evidence that succeeding generations may continue to use healthcare in Turkey in the future, facilitated by European Union legislation and reimbursement from Dutch insurers (9, 10). Studying Dutch residents of Turkish background also has international relevance in view of the large ethnic Turkish populations in other northern European countries, including Germany, France, Austria, Belgium and Denmark (11, 12).

Previous research has primarily examined the immediate motives reported by people for using cross-border healthcare (1). From an individual perspective, former migrants appeared to utilise services in the country of origin on opportunistic occasions, persuaded and guided by their social networks or spurred by a high burden of health problems or unmet needs attributable to language or cultural barriers (3, 7, 13). However, some are also driven by factors relating to both national healthcare systems. They report, for instance, long waiting times, unnecessary delays, limited access to specialist care in their country of residence (3, 7, 13), and rapidity and effectiveness of services in the country of origin (7, 14). Together, experiences like these act as push and pull factors for cross-border healthcare use (14). Studies among people using services in different healthcare systems addressed either the use by migrants of traditional or alternative medicine to complement conventional services within a Western country, or the use of traditional medicine to complement conventional medical services within a non-Western country (15, 16). We are not aware of studies that have used the cultural systems perspective to explore in depth the experiences that people with an immigrant background have with utilising conventional healthcare services in both the country of residence and the country of origin.

For peer review only - http://bmjopen.bmj.com/site/about/guidelines.xhtml

To better understand the motivations and mechanisms that underlie frequent service use in the Dutch and Turkish healthcare systems by Dutch residents of Turkish origin, we designed a qualitative study guided by the cultural systems perspective as proposed by Arthur Kleinman (17). Kleinman regards each healthcare system as a cultural system with its own set of rules and values for dealing with health, illness and healing, even though different systems may share the same foundation of conventional medicine. Such a cultural systems perspective could help us understand the compatibility between a healthcare system and the cultural values of its users by comparing the different systems during the actual transnational use of healthcare by patients.

METHODS

Theoretical framework

Viewing a healthcare system as a cultural system enabled us to compare its cultural rules with the cultural values of its users (17, 18). Differences therein express themselves during healthcare consultations as mismatches between the patient's and the provider's explanatory models of illness. Such models include beliefs about aetiology, symptoms, pathophysiology, and course of sickness and/or treatment. In the case of transnational healthcare use, cultural mismatches between explanatory models are more likely to occur, as a medical consultation is influenced by the rules and values of two national healthcare systems.

Cultural mismatches may occur in all three phases of a consultation (17, 19). In the first phase, a patient presents his or her sensations of illness and tries to persuade the healthcare provider to transform these sensations into symptoms. Core to the second phase is the transition of an illness into a disease, as the healthcare provider, through further

For peer review only - http://bmjopen.bmj.com/site/about/guidelines.xhtml

diagnostics, links the patient's symptoms to a particular disease, syndrome or condition. In the final phase of the consultation, the provider presents a treatment regimen. Each consultation phase thus leads to a specific endpoint: defined symptoms, diagnosis and treatment. These endpoints may be – or may not be – acceptable within the patient's explanatory model of illness.

If we compare the Turkish and Dutch healthcare systems, they appear to generally provide similar quality of care in objective terms, but they differ in the organisation and delivery of services (20). In Turkey, alongside municipal health centres for low-complexity non-acute care, patients have direct access to specialist care (21). In addition, the Turkish healthcare system is interventionist in nature, including a generous use of both the available diagnostics (aimed at 'ruling in' disease) and the available treatment options (22). In the Dutch system, general practitioners provide low-complexity care, while also acting as gatekeepers in deciding if and when referral to specialist care is needed (21). Dutch primary care is non-interventionist in nature, reflected by both its stepwise diagnostics (aimed at 'ruling out' disease) and its stepwise treatment. This wait-and-see approach results in a parsimonious use of screening, diagnostics and drug prescription (21).

In terms of national cultures (23), Turkey has a more hierarchical society than the Netherlands. In such a society, power, authority and control are often centralised (physicians make the decisions, for example). In addition, collectivity is more important in the Turkish than in the Dutch culture. That may imply, for instance, that the family is more involved in the healthcare utilisation trajectory and that the provision of care is more family-based. A final difference in national cultures is the higher level of uncertainty avoidance in Turkey. This means that members of the society feel a greater degree of discomfort with uncertainty

For peer review only - http://bmjopen.bmj.com/site/about/guidelines.xhtml

and ambiguity. This may translate into a stronger desire to *rule out* health risks and potential diseases and to *rule in* some pathophysiological cause to explain sensations or symptoms.

These differences between the Turkish and Dutch healthcare systems and national cultures create a high potential for cultural mismatches in the medical consultations of transnational patients, as they influence which endpoints are reached and are considered acceptable in medical consultations. If left unaddressed, cultural mismatches may heighten the risks of medicalisation and of iatrogenic reactions (18, 19).

Participant recruitment, selection and setting

Two primary care practices in Amsterdam joined the study. Both practices included an ethnic-concordant general practitioner, thereby excluding poor mastery of Dutch as a potential reason for healthcare use in Turkey (1). Patients were identified by their GP as candidate respondents if they came in for consultation after obtaining healthcare in Turkey, and if they generally visited the Dutch practice more than once a month. The GPs were asked to invite a variety of candidates to ensure diversity in terms of gender, education and migration generation. After being notified by the GP, AŞ phoned sixteen Dutch primary care patients with Turkish backgrounds for an interview appointment. Three patients declined participation due to work obligations at possible interview times.

Data collection

The interviews took place from August to November 2018 in a private room at the primary care practice or at the patient’s home, depending on their preference. Some respondents wanted their spouses and/or children to be present in order to help to recollect, supplement and/or clarify their own stories. AŞ made sure that the presence of others was voluntary.

For peer review only - http://bmjopen.bmj.com/site/about/guidelines.xhtml

For peer review only

The interviews ranged from 45 to 120 minutes. They were held primarily in Turkish, although some eventually became mixtures of Turkish and Dutch. All interviews were audiotaped and, during transcription, translated into English by AŞ.

After the interview, a semi-structured questionnaire was administered to assess the healthcare consumption in Turkey and sociodemographic aspects, including self-reported difficulty with the Dutch language.

Interview approach

The biographic-narrative interpretive interview method was used, as it is well suited for retrieving enriched data in the domain of healthcare (24). AŞ started the interview with the following request: ‘Please tell me the story of your life, in the sense of all the events and experiences that have been important to you personally regarding health, sickness and seeking care.’ After the first three interviews, the interviewer began showing respondents a diagram of a human life cycle along with this query, so as to visually guide them in retrieving their stories without interfering in their thought-forming processes. In the second part of the interview, while sticking to the respondent’s order of topics raised and their choice of words used, the interviewer asked the respondent to elaborate on or clarify certain narratives.

Data analysis

In the first phase of the data analysis, AŞ and JH familiarised themselves with the content of the data by reading through the interview transcripts. Next, they composed an initial coding scheme, inspired, first, by the interview method and grounded theory principles and, second, by the above theoretical framework and the literature on cross-border care. In the subsequent phase, AŞ coded the first four interviews. JH read the coded interviews and

For peer review only - http://bmjopen.bmj.com/site/about/guidelines.xhtml

suggested changes to the coding scheme and/or coded segments. These suggestions were discussed and changes were made by consensus, resulting in a final coding scheme (see Supplementary Material I). Using qualitative data analysis software (MaxQDA, Version 2018, VERBI, Berlin), the first author coded all interviews, which were then checked by the second author. Emerging themes and patterns were visualised and discussed together. Both authors deemed data saturation to have been reached after analysing nine interviews.

Research Ethics Approval

The Dutch legislation (the Medical Research Involving Human Subjects Act) does not require ethics approval for this type of interview study. Respondents were asked to sign a written consent form for the use of their recordings (for this study only), to confirm their ownership of their recordings at all times, and to guarantee confidentiality. Data and analysis logs were stored in a protected digital environment, in accordance with the Amsterdam UMC guidelines and Dutch and EU privacy legislation.

Patient and Public Involvement

No patient involved.

Reflexivity

AŞ had extensive training in qualitative research and previous experience with the same community and topic (5). During the interview introduction, he explained his role as both a researcher and a physician. It appeared that respondents tended to consider him a member of the same community who had been successful in developing his potentials in Dutch society. Respondents also seemed proud that he was benefiting the community due to his

For peer review only

choice of a medical career. AŞ was aware that such ascribed authority could lead to socially desirable reporting. He attempted to avoid that by creating an open atmosphere and by staying alert to hesitations in respondents' narratives and any disagreements with his summarisations of their stories. Although all respondents appeared willing to participate in an open and honest fashion, a few initially showed signs of distrust due to the ethnic concordance and the medical profession of the interviewer. They seemed wary of their stories being shared with community members or other health professionals. After reassurance about the anonymity of respondents and their option of halting the interview at all times, all respondents were eventually willing to openly share their intimate narratives.

RESULTS

After describing the respondents' characteristics (Table 1), we highlight the prominent themes that emerged in their narratives on (A) their medical consultations in the Netherlands, (B) their healthcare utilisation in Turkey and (C) their subsequent visits to their Dutch general practitioner. The supporting quotations (Q1-Q20) are presented in Table 2-4.

Respondent characteristics

Our 13 participants were between 39 and 78 years of age; 5 were in paid employment, 6 were born in the Netherlands, and 5 reported difficulty with the Dutch language (Table 1).

[Insert Table 1]

A. Healthcare utilisation in Netherlands

Illness presentation and persuasion

For peer review only - http://bmjopen.bmj.com/site/about/guidelines.xhtml

In the first phase of consultation, respondents felt that their general practitioner chose a wait-and-see approach, and they often did not understand why. Respondents felt that their sensations were either not acknowledged as symptoms, or were interpreted as symptoms giving insufficient reason for further diagnostic investigation to rule out underlying causes. This approach was out of line with the perceived urgency of the respondents' embodied sensations and their suspicions that something was wrong. They felt that both basic physical examination and lab testing were definitely needed to find out the cause (Q01). Many respondents said they tried numerous times to convince their GPs to deviate from the wait-and-see approach, often without success (Q02).

Respondents frequently indicated that the wait-and-see approach did not provide them with an acceptable endpoint. It made them feel they were not taken seriously or not getting recognition as a patient. Many wondered whether they were treated differently to ethnic Dutch patients, and some explicitly insisted that they were. Some attributed the wait-and-see approach to language barriers, but others interpreted it as being treated as a second-class citizen (Q03).

Illness-to-disease transition

In the second phase of the consultation, even if sensations of illness got transformed into symptoms, respondents insisted they still needed proof that the symptoms had a pathophysiological origin. They often did not understand why their Dutch GP failed to use all the available diagnostics to further 'rule in' a cause (Q04).

Stepwise diagnostics did not address respondents' need for risk avoidance and their feeling that something bad could be happening. Especially cancer was believed by patients to be unpredictable in its presentation, pace and mortality, and should therefore be ruled in

For peer review only - http://bmjopen.bmj.com/site/about/guidelines.xhtml

with all the tests available. Hearsay experiences in their social network, where people believed unnecessary harm or death could have been prevented, reinforced the pressing need patients felt to follow-up on their symptoms (Q05).

In such cases, most respondents did not regard the diagnosis offered to be an acceptable endpoint but believed referral to a specialist was needed. However, given the gatekeeper role of Dutch general practitioners, respondents reported they did not get referred easily. While some respondents believed that certain authorities were prohibiting specialist referral, the GP's reluctance made most respondents question the medical knowledge of their doctor (Q06). As a result, respondents said they felt part of an experiment, having to undergo a variety of tests first before receiving proper diagnoses (Q07).

Acceptable treatment regimen

Respondents' stories reflected a conviction that when something is wrong it needs a quick fix. Hence, they did not understand why they needed to try different treatments first before they got the most optimal treatment available that would eradicate their symptoms (Q08). In the respondents' perceptions, being given mere symptom relief instead of treatment for the underlying cause (which they assumed to be present) was no acceptable treatment procedure and just caused unnecessary delay (Q09). Again, respondents said they felt like part of an experiment in which other treatment options were tested out before effective treatment was provided (Q10).

[Insert Table 2]

For peer review only - http://bmjopen.bmj.com/site/about/guidelines.xhtml

For peer review only

B. Healthcare utilisation in Turkey

Provoked by their experiences in the Dutch healthcare system (including perceived lack of recognition, mismatches in explanatory models of illness and the provision of unaccepted diagnoses), many respondents felt a strong need to consult the Turkish system, which they typically effectuated during an already scheduled holiday.

In Turkey, respondents felt that the care provided was more in line with the expectations they had of healthcare services, as they perceived fewer differences between their own explanatory model of illness and that of their Turkish physicians. That made them feel taken seriously, recognised as patients and treated as fully fledged individuals (Q11).

Most of the respondents' narratives reflected a better match between their own explanatory models of illness and those of the Turkish healthcare system (Q12). Direct access to swift specialist diagnostics was seen as a way of shortcutting the stepwise diagnostics of Dutch general practitioners, in accordance with their felt urgency of risk reduction (Q13).

Most respondents reported that more effective curative treatments were provided in Turkey, and they implied that they perceived provision of strong medication or an operation to be the only two decisive curative options for health improvement, thereby shortcutting the stepwise treatment approach of the Dutch healthcare system (Q14).

Most respondents thus saw treatment in Turkey as a valid option. This was limited perhaps by a lack of sufficient holidays and/or lack of a local social network for aftercare, but definitely not by financial restraints, even if specific services were not reimbursable (Q15).

[Insert Table 3]

For peer review only - http://bmjopen.bmj.com/site/about/guidelines.xhtml

C. Healthcare utilisation on return to the Netherlands

Upon their return to the Netherlands, most respondents went back to their Dutch GP to present Turkish test results, diagnoses and/or suggested treatments in order to discuss subsequent steps in the treatment process. This was not always without difficulty (Q16).

In case the consultation endpoint provided in Turkey was accepted by the Dutch healthcare provider, that justified the respondent's considerations for using Turkish health services and helped to compensate for perceived shortcomings in the Dutch system. At the same time, in case Turkish test results were not accepted, similar feelings of justification were expressed, as the fact that something had actually been found was perceived by respondents as comforting (Q17).

Respondents additionally argued that using healthcare in Turkey would be unnecessary and preventable if their GPs would just attend correctly to their health symptoms (Q18). Nonetheless, respondents said they also appreciated the opportunity to discuss the test results and treatment options provided in Turkey upon their return in the Netherlands. As this was seen as a specific quality of Dutch general practitioners – being a case manager for their patients' health and illnesses – the Turkish respondents continued to use primary as well as specialist healthcare in the Netherlands (Q19).

However, sometimes when the Turkish and Dutch systems provided conflicting diagnoses or treatment options, respondents were left in despair, unsure which version to believe. Such respondents nevertheless continued to see transnational healthcare use as one option to find solutions to their health problems (Q20).

[Insert Table 4]

For peer review only - http://bmjopen.bmj.com/site/about/guidelines.xhtml

DISCUSSION

Summery of the findings

This qualitative study among Dutch residents with a Turkish ethnic background showed that: (A) cross-border healthcare use was fostered by cultural mismatches between expected and provided services and by differing explanatory models of illness upheld by patients and Dutch providers; (B) both transnationalism in patients and entitlements to insurance reimbursement facilitated the use of Turkish health services to bypass perceived barriers in the Dutch system; (C) cultural mismatches were reinforced during GP consultations after the patients' return to the Netherlands, thereby inducing further service use abroad.

Interpretation

Our findings indicate that the regular transnational healthcare use of Dutch residents with a Turkish ethnic background (6) may be the result of cultural differences in both healthcare systems and national cultures, as these created mismatches between the patients' and physicians' explanatory models of illness. Although such mismatches made some of our respondents feel a second-class citizen, most of them continued to see their GP as their case manager and a reliable source in seeking care. This means that the GP in the Netherlands, and perhaps also in other counties with a similar gatekeeper system, could play key role in reducing transnational healthcare use. Although cultural gaps may be difficult to bridge (21), cross-border healthcare utilization also rests on individual beliefs, motivation and decisions (15, 16). Therefore, one approach is to strengthen culturally sensitive and competent care during the consultation, as to achieve mutual understanding between the patient and the GP (17, 25). First, this includes being aware of cultural differences, such as in uncertainty avoidance (23), and the related need to find pathophysiological causes of sensations and

For peer review only - http://bmjopen.bmj.com/site/about/guidelines.xhtml

symptoms. Second, it requires cross-cultural communication (25), such as discussing mismatches and negotiating the need for transnational healthcare use (17), including possible iatrogenic outcomes and timely seeking professional help if these might occur (4).

However, providing culturally sensitive care alone may not be sufficient to change the transnational healthcare use of Dutch residents of Turkish origin. Our results confirm that the insurers' reimbursement of healthcare and/or the low cost of care in the country of origin may play an equally important part in transnational healthcare uptake (1, 26). These instruments made opting for health services in Turkey feel natural to our respondents. For Dutch insurance companies, one option to limit the higher than average healthcare use could be contracting only a selection of care providers for the delivery of care to people with Dutch insurance (9). Currently, insurance companies reimburse services from all possible providers abroad, while in the Netherlands they only reimburse care from "preferred providers" meeting certain quality standards (21). Introducing similar quality procedures in countries abroad could also contribute to a further harmonisation between health services in different countries as well as to cost-effective care, while the patient's option to obtain care abroad is being maintained. To additionally minimise the risks of medicalisation and adverse iatrogenic effects, and to coordinate transnational diagnostics and treatment, an online portal for international medical information transfer could be considered.

Strength and limitations

Our main strength is the use of a cultural systems perspective (17, 27). This helped us to see how transnational healthcare use may be fostered by an interaction of factors in both clinical practice, healthcare system and national culture. Another strength lies in the ethnic-

For peer review only - http://bmjopen.bmj.com/site/about/guidelines.xhtml

concordant interviewer (AŞ), who was familiar with respondents' language and cultural expressions. As the biographic-narrative interpretive method (24, 28, 29) led to in-depth and meaningful information, we feel confident to have successfully addressed the potential downsides of such an interviewer, by both reassuring anonymity and letting respondents have ownership of the interviews.

An important limitation is the rather small number of participants in the study, sampled from two primary care practices, in one transnational healthcare context. This limits the possibility to generalize our findings to other Dutch primary care patients of Turkish background as well as to other patient populations, both within and beyond our country. Although the pattern in transnational healthcare use we found was pronounced, additional research in other populations and transnational healthcare contexts seems warranted.

A second limitation is that we only included patients with a Turkish background and no patients of Dutch ethnic origin. Therefore, we cannot say that it are especially Dutch patients of Turkish origin that bypass the general practitioner as to access specialist care. As patient satisfaction can increase when such a bypass option is available (30), it would be interesting to compare our findings with data from patients of Dutch ethnic origin.

Conclusions

Our study uncovered how the transnational healthcare use of Dutch patients with a Turkish background was fostered by cultural mismatches with their Dutch general practitioner. Those mismatches reflected differences in both healthcare systems and national cultures. In order to reduce the unwelcome consequences of transnational healthcare use, measures may include strengthening the provision of culturally sensitive care in the country of

For peer review only - http://bmjopen.bmj.com/site/about/guidelines.xhtml

residence, and restricting the reimbursement of care in the country origin while maintaining the option to obtain care abroad.

For peer review only

For peer review only - http://bmjopen.bmj.com/site/about/guidelines.xhtml

Acknowledgements

We are most grateful for the investments of time and energy made by the respondents in sharing their stories and welcoming the first author into their homes. Without them, this study would not have been possible.

Funding

The first author was a PhD candidate at the Amsterdam UMC, University of Amsterdam, with research funded by a MD-PhD fellowship from the same institution. Grant number: N/A.

For peer review only

Competing interest statement

None declared.

Availability of data and materials

The interview transcripts, the models of each phase of medical consultation, and the MaxQDA project file for the current study are not publicly available, as the respondents' conditions of consent restricted the use of the interviews and the derived data to this research study only. The documents and qualitative data analyses are stored locally in accordance with the guidelines set by the anonymized and by Dutch and European Union privacy legislation.

Authors contributions

anonymized analysed the transcripts and drafted the article. JH read the coded interviews and reflected on the analysis and interpretation of the data. anonymized and anonymized contributed to the conception and design of the study, and anonymized provided the daily

For peer review only - http://bmjopen.bmj.com/site/about/guidelines.xhtml

supervision of the fieldwork. All authors were involved in revising initial drafts of the article.

All authors read and approved the final manuscript.

For peer review only

For peer review only - http://bmjopen.bmj.com/site/about/guidelines.xhtml

REFERENCES

1. Villa-Torres L, González-Vázquez T, Fleming PJ, González-González EL, Infante-Xibille C, Chavez R, et al. Transnationalism and health: A systematic literature review on the use of transnationalism in the study of the health practices and behaviors of migrants. Soc Sci Med. 2017;183:70-9.

2. Portes A. Globalization from below. The rise of transnational communities. Princeton: Princeton University; 1997.

3. Lee JY, Kearns RA, Friesen W. Seeking affective health care: Korean immigrants' use of homeland medical services. Health Place. 2010;16(1):108-15.

4. Moynihan R, G⊘tzsche PC, Heath I, Henry D. Selling sickness: the pharmaceutical industry and disease mongeringCommentary: Medicalisation of risk factors. BMJ. 2002;324(7342):886.

5. Şekercan A, Woudstra AJ, Peters RJG, Lamkaddem M, Akgün S, Essink-Bot ML. Dutch citizens of Turkish origin who utilize healthcare services in Turkey: a qualitative study on motives and contextual factors. BMC Health Serv Res. 2018;18(1):289.

6. Şekercan A, Snijder MB, Peters RJ, Stronks K. Associations between healthcare consumption in country of origin and in country of residence by people with Turkish and Moroccan backgrounds living in the Netherlands: the HELIUS study. Eur J Public Health. 2019;29(4):694-9.

7. Şekercan A, Lamkaddem M, Snijder MB, Peters RJ, Essink-Bot ML. Healthcare consumption by ethnic minority people in their country of origin. Eur J Public Health. 2015;25(3):384-90.

8. Smits FT, Brouwer HJ, Zwinderman AH, Mohrs J, Smeets HM, Bosmans JE, et al. Morbidity and doctor characteristics only partly explain the substantial healthcare

For peer review only - http://bmjopen.bmj.com/site/about/guidelines.xhtml

expenditures of frequent attenders: a record linkage study between patient data and reimbursements data. BMC Family Practice. 2013;14(1):138.

9. Healthcare Insurance Company CZ. Healthcare Insurance Abroad 2019 [Available from: https://www.cz.nl/zorgverzekering/buitenland.

10. European Union. European Health Insurance Card 2019 [Available from: https://ec.europa.eu/social/main.jsp?catId=559.

11. Statistics Netherlands. Statline. Sociodemographic characteristics of the Dutch population 2018 [Available from: https://opendata.cbs.nl/statline/#/CBS/en/.

12. Eurostat. Migrants in Europe. A statistical portrait of the first and second generation. 2011.

13. Wang L, Kwak MJ. Immigration, barriers to healthcare and transnational ties: A case study of South Korean immigrants in Toronto, Canada. Soc Sci Med. 2015;133:340-8.

14. Lokdam N, Kristiansen M, Handlos LN, Norredam M. Use of healthcare services in the region of origin among patients with an immigrant background in Denmark: a qualitative study of the motives. BMC Health Services Research. 2016;16(1):99.

15. Norris P, Fa'alau F, Va'ai C, Churchward M, Arroll B. Navigating Between Illness Paradigms: Treatment Seeking by Samoan People in Samoa and New Zealand. Qualitative Health Research. 2009;19(10):1466-75.

16. Weerasinghe MC, Fernando DN. Paradox in treatment seeking: an experience from rural Sri Lanka. Qual Health Res. 2011;21(3):365-72.

17. Kleinman A. Concepts and a model for the comparison of medical systems as cultural systems. Social Science & Medicine Part B: Medical Anthropology. 1978;12:85-93.

18. Kleinman A, Eisenberg L, Good B. Culture, illness, and care: clinical lessons from anthropologic and cross-cultural research. Ann Intern Med. 1978;88(2):251-8.

For peer review only - http://bmjopen.bmj.com/site/about/guidelines.xhtml

19. Hay MC. Reading Sensations: Understanding the Process of Distinguishing `Fine' from `Sick'. Transcultural Psychiatry. 2008;45(2):198-229.

20. Joint Commision International. JCI-Accredited Organizations 2019 [Available from: https://www.jointcommissioninternational.org/about-jci/accredited-organizations/.

21. Kroneman M, Boerma W, Berg Mvd, Groenewegen P, Jong Jd, Ginneken Ev. The Netherlands: health system review. Health system in transition. Kopenhagen: WHO Regional Office for Europe; 2016.

22. OECD & European Union. Health at a glance: Europe 2017 2017 [Available from: https://www.oecd-ilibrary.org/social-issues-migration-health/health-at-a-glance-2017_health_glance-2017-en.

23. Hofstede G. Culture's consequences: Comparing values, behaviors, institutions and organizations across nations. Thousand Oaks, CA: Sage; 2001.

24. Wengraf T. Qualitative research interviewing: Biographic narrative and semistructured methods. Thousand Oaks, CA: Sage; 2001.

25. Watt K, Abbott P, Reath J. Developing cultural competence in general practitioners: an integrative review of the literature. BMC Family Practice. 2016;17(1):158.

26. Su D, Richardson C, Wen M, Pagán JA. Cross-border utilization of health care: evidence from a population-based study in south Texas. Health Serv Res. 2011;46(3):859-76.

27. Carey JW. Linking Qualitative and Quantitative Methods: Integrating Cultural Factors into Public Health. Qualitative Health Research. 1993;3(3):298-318.

28. Morse JM. The Cultural Sensitivity of Grounded Theory. Qualitative Health Research. 2001;11(6):721-2.

29. Berger R. Now I see it, now I don't: researcher's position and reflexivity in qualitative research. Qualitative Research. 2015;15(2):219-34.

For peer review only - http://bmjopen.bmj.com/site/about/guidelines.xhtml

30. Aoki T, Yamamoto Y, Ikenoue T, Kaneko M, Kise M, Fujinuma Y, et al. Effect of Patient Experience on Bypassing a Primary Care Gatekeeper: a Multicenter Prospective Cohort Study in Japan. J Gen Intern Med. 2018;33(5):722-8.

For peer review only

Table 1. Characteristics of interviewed respondents

	Summarised characteristics		
Participants (number)	13		
Age (range)	39–78		
Gender (% female)	62%		
Migration generation[1] (% 1st, 1.5, 2nd)	31	23	46
Employment status (% paid, % unemployed, % retired)	38	54	8
Educational attainment level[2] (% low, middle, high)	70	30	0
Number of years in Netherlands (% born in NL, range)	31	24–54	
Number of yearly visits to Turkey (range)	0.5–4		
Preference to stay in which country[3] (% NL, % TR, % circular)	23	23	54
Number of diseases (range)	0–6		
Difficulty with Dutch language (% yes)	31		

[1] 1.5 migration generation: migration to Netherlands before age 12

[2] Low: primary or less education; middle: lower general or vocational secondary; high: upper general or vocational secondary or tertiary

[3] Circular: preference to stay longer periods of time in both countries

For peer review only

Table 2. Quotes supporting Results part A. Healthcare utilisation in the Netherlands

Quote no.	Quotation
Q01	My first experience.... I was at work, I'd been tired lately, I only wanted to sleep. I went to my GP. He was my first GP after my own GP retired. He was new, fresh. He didn't even do a physical examination. His answer was, 'Yeah, just keep going. You're young, you don't want to be on social welfare.' I said, 'You don't know me. You didn't even do a medical examination and you don't know what I have.' He told me, 'You should just live with it, there's some things we can't explain.' I said to him, 'What did you do? Nothing.' I went away angry. [I-03]
Q02	This time with my right leg.... Four months ago I had a sudden pain in my right leg. I went to my GP; the pain was of a degree that I couldn't walk. I was stumbling. I went to my GP and said my leg really hurt, it wasn't a normal pain. I couldn't stand, I had to lean on my knee to vacuum the floor. I said to him that the pain in my leg was not a normal pain. He said no, you probably overstrained it. I went to my GP twice, and the third time I went to the emergency department. I went to the ... hospital; on the phone they'd said not to worry about it, just take [painkiller] and it'll go away. I went to the GP for the third time with my daughter *[anger in the voice]*. I said to him there's a torn tissue in my foot, I'm not a doctor but a patient. He didn't believe I had the pain. My ... GP said that if it was torn there would be a bruise. I said this is not a normal pain. He gave me [painkiller] again. [I-01]

For peer review only - http://bmjopen.bmj.com/site/about/guidelines.xhtml

Q03	If it really is a doctor who is discriminating, then you see the attitude immediately. He calls your name and walks back. *[By contrast:]* 'Mister Janssen' *[typical Dutch surname]*.... He waits. 'Hello Mister Janssen, I'm Dr Smit, please come with me.' Stays politely behind that man. The doctor stays behind that man. But when it's a foreigner, whoosh.... You don't see where the doctor's gone to. [I-05]
Q04	What kind of testing did you guys do? … If I have a problem, the answer is take a paracetamol, see how it's going a week later and then we'll check further. A week later, if it didn't work, they do some lab tests, but it's only one or two options they look at. No findings, then again, and again and again. It's tiresome.... Nowadays we have everything, the best machines, but if they're not used properly then what good are they to me? [I-03]
Q05	Why does it take a month in the Netherlands [to get an appointment]? You get cancer. A friend tells you he didn't hear about his for 2 months. Why can't [the testing] be the next day? But it takes a whole month. It might've metastasised by then. These events happened recently, what more can I tell you? There's lots of stories like that. Because I often go as a translator I know what those people experience. [I-09]
Q06	I don't blame the GPs, because they only have certain knowledge. But then I say okay, I can accept that you only possess certain knowledge, I can appreciate that, but if it's beyond your knowledge then send me to a specialist.... If you don't have the knowledge, then you need to refer me. [I-05]

For peer review only - http://bmjopen.bmj.com/site/about/guidelines.xhtml

Q07	She [the GP] told me it's probably gastroenteritis, even though I'd told her I hadn't been eating or drinking for a couple of days and felt like knives were being stabbed in my gut. She didn't do anything. She told me I had to submit a stool sample the following day to the lab, so they can maybe see it's a bacteria. The following day I didn't end up at the lab but in the emergency department, because I fainted. They brought me to the emergency department and they found I was completely dehydrated. The GP hadn't done any checks. I was in hospital for 2 weeks. [I-03]
Q08	For example, I tell them I have stomach aches or that something else hurts. Maybe there's a bacteria. However, they want to do some research [with different treatments] first. First they'll give you a painkiller to try, and if that doesn't work than we can try another one. If you've already had a light medication, why don't they just try prescribing the other medicine that will have an effect? [I-08]
Q09	They only thing they can tell you and do for you is give painkillers and paracetamol, nothing else. In the Netherlands, how can I say it, they only take care of your disease when it has progressed for quite a while. [I-12]
Q10	I got a splint that would slip off after two steps. I went back and got another one. Exactly the same, after two steps it slipped off. I went back again. Listen, what are we up to here? What do you guys want? Am I the first person this happened to, that had to walk around with a splint? After great difficulty ... I got referred.... Listen, why didn't you guys give me that in the first place? Why? It was a mere €10 more.... They don't come through with the full

For peer review only - http://bmjopen.bmj.com/site/about/guidelines.xhtml

	treatment but do everything step by step. I don't mind that. I can understand that they don't give my body an uppercut. I can understand that, but some things like those splints – why do you need to treat me like an experiment? [I-05]

For peer review only

For peer review only - http://bmjopen.bmj.com/site/about/guidelines.xhtml

Table 3. Quotes supporting Results part B. Healthcare utilization in Turkey

Quote no.	Quotation
Q11	I notice when I'm in Turkey that they listen to me.... Okay, these are special clinics you visit, I understand that as well, but there they not only think about their own specialisation, but also outside of that.... It felt more like they took me seriously than they do here. [I-03]
Q12	My [Dutch] GP, I told him something was wrong but I didn't know what. I told him it can't be good for your heart to beat at such a speed. He told me that's what it's made for. No, a heart is made to beat at a certain rhythm and to accelerate when you exercise or get frightened. It was made to function like that. I asked him what will happen if my heart goes on beating this fast? Yeah, then maybe you'll live 50 years instead of 80. You can't give a patient an answer like that. To me that was the limit. So I took a plane to Istanbul and went to a cardiologist because I thought something was wrong with my heart. The doctor thought I'd already had some bloodwork done, but he didn't ask what the results were. I told him they couldn't find anything, but that I have a high heart rhythm and I'm tired. He did all the diagnostics a cardiologist would do: an ultrasound, an exercise stress test, a bicycle test, a lab workup. In the ultrasound they looked at the blood supply in my heart, looked for hardening of the arteries. Everything he told me put me at ease. He told me he could find nothing. He looked at my lab workup – he did a full workup – and he told me the problem was clear. He said they probably didn't test that. I had a vitamin D deficiency. [I-03]

For peer review only - http://bmjopen.bmj.com/site/about/guidelines.xhtml

Q13	In the Netherlands they suspected my husband's pancreas, he was admitted to hospital, he had lost a lot of weight and they'd given him an appointment for a month later. He went to Turkey, got his results, went back and gave them the report. The answer he got was that they [the Dutch hospital] were also planning to look at that. But it takes a whole month, maybe it could have metastasised by then? [I-09]
Q14	In Turkey they tell me I need an operation for my most recent symptoms. Even if they tell me no in the Netherlands, I'll go to Turkey for the necessary [operation]. Nothing more I can do. [I-02]
Q15	This year I was admitted to hospital [in Turkey] and they [Dutch insurance company] told me they might not pay for it. That doesn't interest me. Just let me know what's wrong and what we can do about it. I want to be free of the pain.... An MRI is just 400 lira, €50. I won't die from that expense, but getting an MRI in the Netherlands? ... I also bring back a lot of medicines from Turkey nowadays. [I-03]

For peer review only - http://bmjopen.bmj.com/site/about/guidelines.xhtml

Table 4. Quotes supporting Results part C. Healthcare utilisation on return to the Netherlands

Quote no.	Quotation
Q16	I had great difficulty convincing my GP that this was it. Couldn't be possible! I showed him the blood levels. I don't understand them, but according to my GP my vitamin B was above the Dutch recommended level. But the professor in Turkey had told me ... people from Turkey should have above 100.... I told my GP, listen, this and that is the case. She wouldn't believe it. But all right, it didn't matter, with great difficulty they gave me those 3 injections, once a week, and thank God, the symptoms were gone. [I-05]
Q17	It's not nice to not be heard, that's what I notice in the Netherlands, be it a GP or a medical specialist. They're too simplistic about your issues, they send you away too easily and there's no alternative but to think up another solution.... Last time, my reassurance in Turkey was only because they do extensive testing. And even if it eventually turns out to be something small, they can explain my health issue with it. And if you treat that, it's over after that. That's my experience, that's why I prefer using healthcare in my country of origin. Nowadays I just buy a ticket without hesitating. My second opinion is now in Turkey. [I-03]
Q18	I had to go 3000 kilometres to learn that I have a vitamin D deficiency. No answer. 'We didn't think of that.' I told them my body was giving a signal. They told me it was all in my mind. I got vitamin D drops. I noticed my heart rhythm was decreasing. So that was the cause of my problem. You're

For peer review only - http://bmjopen.bmj.com/site/about/guidelines.xhtml

	inclined to start searching elsewhere. In the past 10 to 15 years I've been doing that more and more. [I-03]
Q19	Also about my legs and sleeplessness. It had taken me years, and in Turkey the whole diagnosis took 2 hours and they told me I have restless legs. True, the whole night. He told me to take these pills, and thank God I still have those pills in case I need them. I went to the GP in the Netherlands and it all started up again from the beginning. Eventually she accepted it, but it had some side-effects. So again I'm happy with my GP, I can get along well with her, and we're now trying the third medication because of the side-effects.... Now when I get side-effects, we try something else. That's also positive... [I-05]
Q20	If everything is fine, why do I have problems? I'm still suffering.... I'm at the point where I am losing myself. They couldn't find a solution to my headaches. For years, I've been going to doctors here as well as in Turkey. If need be I'll just have to go in Turkey as well. [I-02]

For peer review only - http://bmjopen.bmj.com/site/about/guidelines.xhtml

Supplementary Material 1. Codes used in the Qualitative Analysis

Steps of consultation (negotiation at each phase)	
	Illness presentation and persuading
	illness to disease translation
	Acceptable treatment regimen
Sensation to symptom	
	Vulnerability
	Urgency
	Disability/death (seriousness)
Core adaptive tasks of healthcare systems	
	Dealing with illness
	Healthcare strategies
	Consultation
	Healing activities
	Health influencing behaviours
	Dealing with healthcare outcomes
Explanatory model	
	Patient
	Provider
	Differences/troubles
	Agreement
Endpoint	
	Conflicting
	Unacceptable
	Acceptable
	Test result
	Diagnosis
	It's nothing serious
Turkish Healthcare	
	Direct access to specialist care
	Ruling in versus ruling out
	Interventionalist
	Value for money
	"Broad" & fast diagnostics
Dutch Healthcare	
	Specialist referral
	Ruling out versus ruling in
	Non-invertentionalist
	Stepwise diagnosis
	Stepwise treatment
Life World Intern	
	Turkish examples
	Dutch examples
	Cultural assumptions
Life World Extern	

For peer review only - http://bmjopen.bmj.com/site/about/guidelines.xhtml

Dutch setting experience
Turkish setting experience
Cultural context
Being an experiment
Self-reliance
Finances
Language
Delay
Shopping (behaviour)
Uncertainty avoidance
Transnationalism
Access without barriers<br>Why? Expressing lack of understanding<br>Metaphor

For peer review only

For peer review only - http://bmjopen.bmj.com/site/about/guidelines.xhtml

Consolidated criteria for reporting qualitative studies (COREQ): 32-item checklist

Developed from: Tong A, Sainsbury P, Craig J. Consolidated criteria for reporting qualitative research (COREQ): a 32-item checklist for interviews and focus groups. *International Journal for Quality in Health Care*. 2007. Volume 19, Number 6: pp. 349 – 357

YOU MUST PROVIDE A RESPONSE FOR ALL ITEMS. ENTER N/A IF NOT APPLICABLE

No. Item	Guide questions/description	Reported on Page #
Domain 1: Research team and reflexivity		
Personal Characteristics		
1. Inter viewer/facilitator	Which author/s conducted the interview or focus group?	9
2. Credentials	What were the researcher's credentials? E.g. PhD, MD	1
3. Occupation	What was their occupation at the time of the study?	10-11
4. Gender	Was the researcher male or female?	10-11
5. Experience and training	What experience or training did the researcher have?	10-11
Relationship with participants		
6. Relationship established	Was a relationship established prior to study commencement?	8, 10-11
7. Participant knowledge of the interviewer	What did the participants know about the researcher? e.g. personal goals, reasons for doing the research	10-11
8. Interviewer characteristics	What characteristics were reported about the inter viewer/facilitator? e.g. Bias, assumptions, reasons and interests in the research topic	10-11
Domain 2: study design		
Theoretical framework		
9. Methodological orientation and Theory	What methodological orientation was stated to underpin the study? e.g. grounded theory, discourse analysis, ethnography, phenomenology, content analysis	6-8, 9
Participant selection		
10. Sampling	How were participants selected? e.g. purposive, convenience, consecutive, snowball	8
11. Method of approach	How were participants approached? e.g. face-to-face, telephone, mail, email	8
12. Sample size	How many participants were in the study?	8
13. Non-participation	How many people refused to participate or dropped out? Reasons?	8
Setting		
14. Setting of data collection	Where was the data collected? e.g. home, clinic, workplace	8
15. Presence of non-participants	Was anyone else present besides the participants and researchers?	8-9
16. Description of sample	What are the important characteristics of the sample? e.g. demographic data, date	11, 25

For peer review only - http://bmjopen.bmj.com/site/about/guidelines.xhtml

Data collection		
17. Interview guide	Were questions, prompts, guides provided by the authors? Was it pilot tested?	9
18. Repeat interviews	Were repeat interviews carried out? If yes, how many?	N/A
19. Audio/visual recording	Did the research use audio or visual recording to collect the data?	8
20. Field notes	Were field notes made during and/or after the interview or focus group?	N/A
21. Duration	What was the duration of the interviews or focus group?	8
22. Data saturation	Was data saturation discussed?	10
23. Transcripts returned	Were transcripts returned to participants for comment and/or correction?	N/A
Domain 3: analysis and findings		
Data analysis		
24. Number of data coders	How many data coders coded the data?	9-10
25. Description of the coding tree	Did authors provide a description of the coding tree?	9-10; Supplementary Material I
26. Derivation of themes	Were themes identified in advance or derived from the data?	9-10
27. Software	What software, if applicable, was used to manage the data?	9-10
28. Participant checking	Did participants provide feedback on the findings?	N/A
Reporting		
29. Quotations presented	Were participant quotations presented to illustrate the themes/findings? Was each quotation identified? e.g. participant number	26-33
30. Data and findings consistent	Was there consistency between the data presented and the findings?	11-15, 26-33
31. Clarity of major themes	Were major themes clearly presented in the findings?	11-15
32. Clarity of minor themes	Is there a description of diverse cases or discussion of minor themes?	11-15

Once you have completed this checklist, please save a copy and upload it as part of your submission. When requested to do so as part of the upload process, please select the file type: *Checklist*. You will NOT be able to proceed with submission unless the checklist has been uploaded. Please DO NOT include this checklist as part of the main manuscript document. It must be uploaded as a separate file.

For peer review only

For peer review only - http://bmjopen.bmj.com/site/about/guidelines.xhtml

BMJ Open

Understanding transnational healthcare use in immigrant communities from a cultural systems perspective: A qualitative study of Dutch residents with a Turkish background

Journal:	*BMJ Open*
Manuscript ID	bmjopen-2021-051903.R1
Article Type:	Original research
Date Submitted by the Author:	29-Jun-2021
Complete List of Authors:	Şekercan, Aydin; Amsterdam UMC Locatie AMC, Public and Occupation Health<br>Harting, Janneke; Amsterdam UMC Locatie AMC,<br>Peters, Ron; Amsterdam UMC - Locatie AMC, Cardiology<br>Stronks, Karien; Amsterdam UMC Locatie AMC, Public and Occupational Health
**Primary Subject Heading**:	Public health
Secondary Subject Heading:	Public health, General practice / Family practice, Patient-centred medicine
Keywords:	PRIMARY CARE, PUBLIC HEALTH, QUALITATIVE RESEARCH

SCHOLARONE™
Manuscripts

For peer review only - http://bmjopen.bmj.com/site/about/guidelines.xhtml

I, the Submitting Author has the right to grant and does grant on behalf of all authors of the Work (as defined in the below author licence), an exclusive licence and/or a non-exclusive licence for contributions from authors who are: i) UK Crown employees; ii) where BMJ has agreed a CC-BY licence shall apply, and/or iii) in accordance with the terms applicable for US Federal Government officers or employees acting as part of their official duties; on a worldwide, perpetual, irrevocable, royalty-free basis to BMJ Publishing Group Ltd ("BMJ") its licensees and where the relevant Journal is co-owned by BMJ to the co-owners of the Journal, to publish the Work in this journal and any other BMJ products and to exploit all rights, as set out in our licence.

The Submitting Author accepts and understands that any supply made under these terms is made by BMJ to the Submitting Author unless you are acting as an employee on behalf of your employer or a postgraduate student of an affiliated institution which is paying any applicable article publishing charge ("APC") for Open Access articles. Where the Submitting Author wishes to make the Work available on an Open Access basis (and intends to pay the relevant APC), the terms of reuse of such Open Access shall be governed by a Creative Commons licence – details of these licences and which Creative Commons licence will apply to this Work are set out in our licence referred to above.

Other than as permitted in any relevant BMJ Author's Self Archiving Policies, I confirm this Work has not been accepted for publication elsewhere, is not being considered for publication elsewhere and does not duplicate material already published. I confirm all authors consent to publication of this Work and authorise the granting of this licence.

For peer review only

TITLE

Understanding transnational healthcare use in immigrant communities from a cultural systems perspective: A qualitative study of Dutch residents with a Turkish background

AUTHORS

Aydın Şekercan[1,2], Janneke Harting[1], Ron J. Peters[3], Karien Stronks[1]

AFFILIATIONS

1 Amsterdam UMC, University of Amsterdam, Department of Public and Occupational Health, Amsterdam Public Health research institute, Amsterdam, the Netherlands

2 Dutch hospital "Onze Lieve Vrouwe Gasthuis", location East, Department of General Surgery, Amsterdam, the Netherlands

3 Amsterdam UMC, University of Amsterdam, Department of Cardiology, Amsterdam, the Netherlands

For peer review only - http://bmjopen.bmj.com/site/about/guidelines.xhtml

WORD COUNT

Main text: 6.679 [of which 4.567 are text; and 1.930 are quotes]

CORRESPONDING AUTHOR

Janneke Harting

Amsterdam UMC, Department of Public and Occupational Health

Meibergdreef 15, 1105 AZ Amsterdam, the Netherlands

j.harting@amsterdamumc.nl

+31 20 566 50 49

For peer review only

ABSTRACT

Objectives

Transnational utilisation of healthcare by people with an immigrant background carries risks, including medicalisation and adverse iatrogenic outcomes. We investigated the drivers behind such transnational healthcare use from a cultural perspective on health systems.

Design

Qualitative interview study (2018).

Setting

Two primary care practices in Amsterdam, the Netherlands.

Participants

Thirteen Dutch patients of Turkish background, who had obtained healthcare in Turkey, and who in general visited the primary care practice more than once a month.

Results

In the respondents' stories, we observed how: (A) cross-border healthcare use was encouraged by cultural mismatches between expected and provided services and by

For peer review only

For peer review only - http://bmjopen.bmj.com/site/about/guidelines.xhtml

differing explanatory models of illness upheld by patients and Dutch providers; (B) both transnationalism in patients and entitlements to insurance reimbursement facilitated the use of Turkish health services to bypass perceived barriers in the Dutch system; (C) cultural mismatches were reinforced during GP consultations after the patients' return to the Netherlands, thereby inducing further service use abroad.

Conclusions

Although cultural system influences are difficult to bridge, measures to reduce the unwelcome consequences of transnational healthcare use may include (A) strengthening the provision of culturally sensitive care in the country of residence and (B) restricting the reimbursement of care in the country of origin while maintaining the option to obtain care abroad.

KEY TERMS

migrants, cultural system, transnationalism, cross-border care, transnational healthcare

ARTICLE SUMMARY

For peer review only - http://bmjopen.bmj.com/site/about/guidelines.xhtml

Strengths and limitations of the study

- A first strength is our innovative approach to explore the drivers behind transnational healthcare use from a cultural systems perspective
- This cultural systems perspective helped us to understand how transnational healthcare use may be encouraged by an interaction of factors in both clinical practice, healthcare system and national culture
- A second strength is the ethnic-concordant interviewer, familiar with the respondents' language and cultural expressions, and the biographic-narrative interpretive interview method, together leading to in-depth and meaningful information
- An important limitation is the rather small number of participants in the study, sampled from two primary care practices in one transnational healthcare context, which limits the generalizability of our finding to other populations and contexts

For peer review only - http://bmjopen.bmj.com/site/about/guidelines.xhtml

BACKGROUND

Across the globe, the transnational ties of former migrants and their families are enabling them to retain and pursue private, cultural and economic interests in their countries of origin, including the domain of healthcare. This phenomenon is known as transnationalism (1, 2). People with an immigrant background tend to utilise similar healthcare services in both their country of origin and their country of residence (1, 3). High transnational healthcare use may increase the risk of medicalisation, defined as expansion of the medical domain (4). Different diagnostic approaches may cause more ailments to be labelled as diseases (4) and thus also increase the risk of iatrogenic complications resulting from additional treatments (5, 6). Due to a lack of cross-border medical information transfers, upon the patients' return to their country of residence they may be subject to repetitions of diagnostics, drug interactions in treatments and inadequate treatment aftercare (7). In addition, transnational healthcare use is likely to foster antimicrobial drug resistance (6). At the system level, it may increase healthcare spending in the country of residence, pull resources from the public sector in the country of origin, and thus widen health disparities (6).

Motivators for treatment abroad generally include low costs, short waiting lists, quality

For peer review only - http://bmjopen.bmj.com/site/about/guidelines.xhtml

of care and the available medical procedures (8, 9). Yet, the drivers that underlie frequent transnational healthcare use are complex and deserve further investigation(9), as to curtail its adverse effects.

People of Turkish ethnic origin who are resident in the Netherlands are a relevant group for further study of this phenomenon. With about 425.000 people, they form the largest ethnic minority group in the Netherlands (10). Migration from Turkey was encouraged in the 1960s and early 1970s to fill labour shortages in unskilled occupation (11). In the years that followed (1970-80), many “guest workers” brought their spouses and children to the Netherlands (11). As 80% of the Dutch residents of Turkish background identify as being both Dutch and Turkish and maintain ties with the Turkish culture, they can readily compare, utilise and evaluate various features of health services in both countries (2, 7). Being residents of the European Union, they have access to necessary health care in all countries in Europe (12) and Dutch insurance companies reimburse public as well as private healthcare in both the Netherlands and Turkey (13). There is also evidence that succeeding generations may continue to use healthcare in Turkey in the future (12, 13). Studying Dutch residents of Turkish background also has international relevance in view of the large

For peer review only - http://bmjopen.bmj.com/site/about/guidelines.xhtml

ethnic Turkish populations in other northern European countries, including Germany, France, Austria, Belgium and Denmark (10, 14).

Transnational health service use is highly prevalent in Dutch residents with a Turkish background. They are more likely to utilise health services in the country of origin than other migration groups – 46%, as compared to 18% of residents with a Moroccan background (15). This happens even though apparently equivalent facilities are available in the Netherlands (16). Moreover, those who frequently use health services in Turkey also tend to be frequent healthcare users in the Netherlands (i.e. often in the top 10% of frequent users). Their overall rates of utilisation are also high in comparison with their co-ethnics who do not obtain healthcare in Turkey (Dutch primary care use: 40.4% versus 22.5%; specialist medical care use: 57.8% versus 33.9%) (15, 17). The few factors identified to be associated with healthcare use in the country of origin are poorer self-reported health and a wider perceived cultural distance to the healthcare system in the country of residence (16).

Previous research has primarily examined the immediate motives reported by people for using cross-border healthcare (1). From an individual perspective, former

For peer review only - http://bmjopen.bmj.com/site/about/guidelines.xhtml

migrants appeared to utilise services in the country of origin on opportunistic occasions, persuaded and guided by their social networks or spurred by a high burden of health problems or unmet needs attributable to language or cultural barriers (3, 16, 18). However, some are also driven by factors relating to both national healthcare systems. They report, for instance, long waiting times, unnecessary delays, limited access to specialist care in their country of residence (3, 16, 18), and rapidity and effectiveness of services in the country of origin (16, 19). Together, experiences like these act as push and pull factors for cross-border healthcare use (19). Studies among people using services in different healthcare systems addressed either the use by migrants of traditional or alternative medicine to complement conventional services within a Western country, or the use of traditional medicine to complement conventional medical services within a non-Western country (20, 21). We are not aware of studies that have used the cultural systems perspective to explore in depth the experiences that people with an immigrant background have with utilising conventional healthcare services in both the country of residence and the country of origin.

For peer review only

To better understand the motivations and mechanisms that underlie frequent service use in the Dutch and Turkish healthcare systems by Dutch residents of Turkish origin, we designed a qualitative study guided by the cultural systems perspective as proposed by Arthur Kleinman (22). Kleinman regards each healthcare system as a cultural system with its own set of rules and values for dealing with health, illness and healing, even though different systems may share the same foundation of conventional medicine. Such a cultural systems perspective could help us understand the compatibility between a healthcare system and the cultural values of its users by comparing the different systems during the actual transnational use of healthcare by patients.

METHODS

Analytical framework

Viewing a healthcare system as a cultural system enabled us to compare its cultural rules with the cultural values of its users (22, 23). Differences therein express themselves during healthcare consultations as mismatches between the patient‘s and the provider‘s explanatory models of illness. Such models include beliefs about

For peer review only - http://bmjopen.bmj.com/site/about/guidelines.xhtml

aetiology, symptoms, pathophysiology, and course of sickness and/or treatment. In the case of transnational healthcare use, cultural mismatches between explanatory models are more likely to occur, as a medical consultation is influenced by the rules and values of two national healthcare systems and two national cultures. Therefore, the analytical framework comprised the concept of cultural mismatches, the differences between national health care systems, and a comparison of national cultures.

Cultural mismatches may occur in all three phases of a consultation (22, 24). In the first phase, a patient presents his or her sensations, as the basis of the recognition that something is wrong, and tries to persuade the healthcare provider to transform these sensations into symptoms, as a recognized objective clinical reality (24). Core to the second phase is the transition of an illness into a disease, once the healthcare provider, through further diagnostics, links the patient's symptoms to a particular disease, syndrome or condition. In the final phase of the consultation, the provider presents a treatment regimen. Each consultation phase thus leads to a specific endpoint: defined symptoms, diagnosis and treatment. These endpoints may be – or may not be – acceptable within the patient's explanatory model of illness.

For peer review only - http://bmjopen.bmj.com/site/about/guidelines.xhtml

If we compare the Turkish and Dutch healthcare systems, they appear to generally provide similar quality of care in objective terms, but they differ in the organisation and delivery of services (25). In Turkey, alongside municipal health centres for low-complexity non-acute care, patients have direct access to specialist care (26). In addition, the Turkish healthcare system is interventionist in nature, including a generous use of both the available diagnostics (aimed at ‘ruling in’ disease) and the available treatment options (27). In the Dutch system, general practitioners provide low-complexity care, while also acting as gatekeepers in deciding if and when referral to specialist care is needed (26). Dutch primary care is non-interventionist in nature, reflected by both its stepwise diagnostics (aimed at ‘ruling out’ disease) and its stepwise treatment. This wait-and-see approach results in a parsimonious use of screening, diagnostics and drug prescription (26).

In terms of national cultures, Turkey has a more hierarchical society than the Netherlands (28). In such a society, power, authority and control are often centralised (physicians make the decisions, for example). In addition, collectivity is more important in the Turkish than in the Dutch culture(28). That may imply, for instance, that the family is more involved in the healthcare utilisation trajectory and that the

For peer review only - http://bmjopen.bmj.com/site/about/guidelines.xhtml

provision of care is more family-based. A final difference in national cultures is the higher level of uncertainty avoidance in Turkey (28). This means that members of the society feel a greater degree of discomfort with uncertainty and ambiguity. This may translate into a stronger desire to *rule out* health risks and potential diseases and to *rule in* some pathophysiological cause to explain sensations or symptoms. Despite these differences between the two nations, it should be noted that culture is a dynamic concept, that may express itself in different ways.

These differences between the Turkish and Dutch healthcare systems and national cultures create a high potential for cultural mismatches in the medical consultations of transnational patients, as they influence which endpoints are reached and which are considered acceptable in medical consultations. If left unaddressed, cultural mismatches may heighten the risks of medicalisation and of iatrogenic reactions (23, 24).

Participant recruitment, selection and setting

Two primary care practices in Amsterdam joined the study. Both practices included an ethnic-concordant general practitioner, thereby excluding poor mastery of Dutch

For peer review only - http://bmjopen.bmj.com/site/about/guidelines.xhtml

as a potential reason for healthcare use in Turkey (1). Patients were identified by their GP as candidate respondents if they came in for consultation after obtaining healthcare in Turkey, and if they generally visited the Dutch practice more than once a month. The GPs were asked to invite a variety of candidates to ensure diversity in terms of gender, education and migration generation. After being notified by the GP, AȘ phoned sixteen Dutch primary care patients with Turkish a background for an interview appointment. Three patients declined participation due to work obligations at possible interview times.

Data collection

The interviews took place from August to November 2018 in a private room at the primary care practice or at the patient's home, depending on their preference. Some respondents wanted their spouses and/or children to be present in order to help to recollect, supplement and/or clarify their own stories. AȘ made sure that the presence of others was voluntary. The interviews ranged from 45 to 120 minutes. They were held primarily in Turkish, although some eventually became mixtures of

For peer review only - http://bmjopen.bmj.com/site/about/guidelines.xhtml

Turkish and Dutch. All interviews were audiotaped and, during transcription, translated into English by AȘ.

After the interview, a semi-structured questionnaire was administered to assess the healthcare consumption in Turkey and sociodemographic aspects, including self-reported difficulty with the Dutch language.

Interview approach

The biographic-narrative interpretive interview method was used, as it is well suited for retrieving enriched data in the domain of healthcare (29). AȘ started the interview with the following request: 'Please tell me the story of your life, in the sense of all the events and experiences that have been important to you personally regarding health, sickness and seeking care.' In the first three interviews the respondents tended to focus on one episode of healthcare use or to discuss different episodes while mixing these up. Therefore, in the following interviews the interviewer began showing respondents a diagram of a human life cycle, so as to visually guide them in retrieving their stories in a chronological order without interfering in their thought-forming processes. In the second part of the interview, while sticking to the

For peer review only - http://bmjopen.bmj.com/site/about/guidelines.xhtml

respondent's order of topics raised and their choice of words used, the interviewer asked the respondent to elaborate on or clarify certain narratives, specifically in relation to the different phases of the consultation. For the interview guide, please see Supplementary Material I.

Data analysis

In the first phase of the data analysis, AȘ and JH familiarised themselves with the content of the data by reading through the interview transcripts. Next, they composed an initial coding scheme, inspired first by the interview method and grounded theory principles, and second by the above analytical framework and the literature on cross-border care. In the subsequent phase, AȘ coded the first four interviews. JH read the coded interviews and suggested changes to the coding scheme and/or coded segments. These suggestions were discussed and changes were made by consensus, resulting in a final coding scheme (see Supplementary Material II). Using qualitative data analysis software (MaxQDA, Version 2018, VERBI, Berlin), the first author coded all interviews, which were then checked by the second author. Emerging themes and patterns were visualised and discussed together. The pattern

For peer review only - http://bmjopen.bmj.com/site/about/guidelines.xhtml

of drivers behind transnational health care use was almost homogeneous across the sample. Both authors deemed data saturation to have been reached after analysing nine interviews.

Informed consent

Respondents were asked to sign a written consent form for the use of their recordings (for this study only), to confirm their ownership of their recordings at all times, and to guarantee confidentiality.

Patient and Public Involvement

No patients or public involved.

Reflexivity

AȘ had extensive training in qualitative research and previous experience with the same community and topic (7). During the interview introduction, he explained his role as both a researcher and a physician. It appeared that respondents tended to consider him a member of the same community who had been successful in

For peer review only - http://bmjopen.bmj.com/site/about/guidelines.xhtml

developing his potentials in Dutch society. Respondents also seemed proud that he was benefiting the community due to his choice of a medical career. AȘ was aware that such ascribed authority could lead to socially desirable reporting. He attempted to avoid that by creating an open atmosphere and by staying alert to hesitations in respondents' narratives and any disagreements with his summarisations of their stories. Although all respondents appeared willing to participate in an open and honest fashion, a few initially showed signs of distrust due to the ethnic concordance and the medical profession of the interviewer. They seemed wary of their stories being shared with community members or other health professionals. After reassurance about the anonymity of respondents and their option of halting the interview at all times, all respondents were eventually willing to openly share their intimate narratives.

RESULTS

After describing the respondents' characteristics (Table 1), we highlight the prominent themes that emerged in their narratives on (A) their medical consultations

For peer review only - http://bmjopen.bmj.com/site/about/guidelines.xhtml

For peer review only

in the Netherlands, (B) their healthcare utilisation in Turkey and (C) their subsequent visits to their Dutch general practitioner.

Respondent characteristics

Our 13 participants were between 39 and 78 years of age; 5 were in paid employment, 6 were born in the Netherlands, and 5 reported difficulty with the Dutch language (Table 1). Their primary reason to go to Turkey was visiting relatives, while decisions about using healthcare were in general made after arriving in Turkey. Respondents usually made use of private healthcare services, as these were regarded more accessible and providing quicker services than public services.

[Insert Table 1]

A. Healthcare utilisation in Netherlands

Illness presentation and persuasion

In the first phase of consultation, respondents typically felt that their general practitioner chose a wait-and-see approach, and they often did not understand why.

For peer review only - http://bmjopen.bmj.com/site/about/guidelines.xhtml

They felt that their sensations were either not acknowledged as symptoms, or were interpreted as symptoms giving insufficient reason for further diagnostic investigation to rule out underlying causes. This approach was out of line with the perceived urgency of the respondents' embodied sensations and their suspicions that something was wrong. All but one of the respondents explained that both basic physical examination and lab testing were definitely needed to find out the cause (Q01). The vast majority said they tried numerous times to convince their GPs to deviate from the wait-and-see approach, often without success (Q02).

> Q01: My first experience.... I was at work, I'd been tired lately, I only wanted to sleep. I went to my GP. He was my first GP after my own GP retired. He was new, fresh. He didn't even do a physical examination. His answer was, 'Yeah, just keep going. You're young, you don't want to be on social welfare.' I said, 'You don't know me. You didn't even do a medical examination and you don't know what I have.' He told me, 'You should just live with it, there's some things we can't explain.' I said to him, 'What did you do? Nothing.' I went away angry. [I-03, 1rst generation migrant, paid work, no difficulty with Dutch language]

For peer review only - http://bmjopen.bmj.com/site/about/guidelines.xhtml

Q02: This time with my right leg.... Four months ago I had a sudden pain in my right leg. I went to my GP; the pain was of a degree that I couldn't walk. I was stumbling. I went to my GP and said my leg really hurt, it wasn't a normal pain. I couldn't stand, I had to lean on my knee to vacuum the floor. I said to him that the pain in my leg was not a normal pain. He said no, you probably overstrained it. I went to my GP twice, and the third time I went to the emergency department. I went to the ... hospital; on the phone they'd said not to worry about it, just take [painkiller] and it'll go away. I went to the GP for the third time with my daughter *[anger in the voice]*. I said to him there's a torn tissue in my foot, I'm not a doctor but a patient. He didn't believe I had the pain. My ... GP said that if it was torn there would be a bruise. I said this is not a normal pain. He gave me [painkiller] again.

[I-01, 1rst generation migrant, paid work, no difficulty with Dutch language]

Respondents frequently indicated that the wait-and-see approach did not provide them with an acceptable endpoint. It made them feel they were not taken seriously or

For peer review only - http://bmjopen.bmj.com/site/about/guidelines.xhtml

not getting recognition as a patient. Most wondered whether they were treated differently to ethnic Dutch patients, and some explicitly insisted that they were. Some attributed the wait-and-see approach to language barriers, but others interpreted it as being treated as a second-class citizen (Q03).

> Q03: If it really is a doctor who is discriminating, then you see the attitude immediately. He calls your name and walks back. *[By contrast:]* 'Mister Janssen' *[typical Dutch surname]*.... He waits. 'Hello Mister Janssen, I'm Dr Smit, please come with me.' Stays politely behind that man. The doctor stays behind that man. But when it's a foreigner, whoosh.... You don't see where the doctor's gone to.
>
> [I-05, 2nd generation migrant, paid work, no difficulty with Dutch language]

Illness-to-disease transition

In the second phase of the consultation, even if sensations of illness got transformed into symptoms, respondents typically insisted they still needed proof that the

For peer review only - http://bmjopen.bmj.com/site/about/guidelines.xhtml

symptoms had a pathophysiological origin. They often did not understand why their Dutch GP failed to use all the available diagnostics to further ‘rule in’ a cause (Q04).

> Q04: What kind of testing did you guys do? … If I have a problem, the answer is take a paracetamol, see how it’s going a week later and then we’ll check further. A week later, if it didn’t work, they do some lab tests, but it’s only one or two options they look at. No findings, then again, and again and again. It’s tiresome.... Nowadays we have everything, the best machines, but if they’re not used properly then what good are they to me?
>
> [I-03, 1rst generation migrant, paid work, no difficulty with Dutch language]

Generally, stepwise diagnostics did not address the respondents’ need for risk avoidance and their feeling that something bad could be happening. Especially cancer was believed by almost all patients to be unpredictable in its presentation, pace and mortality, and should therefore be ruled in with all the tests available. Hearsay experiences in their social network, where people believed unnecessary

For peer review only

For peer review only - http://bmjopen.bmj.com/site/about/guidelines.xhtml

harm or death could have been prevented, reinforced the pressing need most patients felt to follow-up on their symptoms (Q05).

> Q05: Why does it take a month in the Netherlands [to get an appointment]? You get cancer. A friend tells you he didn't hear about his for 2 months. Why can't [the testing] be the next day? But it takes a whole month. It might've metastasised by then. These events happened recently, what more can I tell you? There's lots of stories like that. Because I often go as a translator I know what those people experience.
>
> [I-09, 2nd generation migrant, unemployed, no difficulty with Dutch language]

In such cases, most respondents did not regard the diagnosis offered to be an acceptable endpoint but believed referral to a specialist was needed. However, given the gatekeeper role of Dutch general practitioners, respondents reported they did not get referred easily. While a few respondents believed that certain authorities were prohibiting specialist referral, the GP's reluctance made most respondents question the medical knowledge of their doctor (Q06). As a result, some respondents said they

For peer review only - http://bmjopen.bmj.com/site/about/guidelines.xhtml

felt part of an experiment, having to undergo a variety of tests first before receiving proper diagnoses (Q07).

Q06: I don't blame the GPs, because they only have certain knowledge. But then I say okay, I can accept that you only possess certain knowledge, I can appreciate that, but if it's beyond your knowledge then send me to a specialist…. If you don't have the knowledge, then you need to refer me. [I-05, 2nd generation migrant, paid work, no difficulty with Dutch language]

Q07: She [the GP] told me it's probably gastroenteritis, even though I'd told her I hadn't been eating or drinking for a couple of days and felt like knives were being stabbed in my gut. She didn't do anything. She told me I had to submit a stool sample the following day to the lab, so they can maybe see it's a bacteria. The following day I didn't end up at the lab but in the emergency department, because I fainted. They brought me to the emergency department and they found I was completely dehydrated. The GP hadn't done any checks. I was in hospital for 2 weeks.

For peer review only - http://bmjopen.bmj.com/site/about/guidelines.xhtml

For peer review only

[I-03, 1rst generation migrant, paid work, no difficulty with Dutch language]

Acceptable treatment regimen

All respondents' stories reflected a conviction that when something is wrong it needs a quick fix. Hence, they did not understand why they needed to try different treatments first before they got the most optimal treatment available that would eradicate their symptoms (Q08). In the respondents' perceptions, being given mere symptom relief instead of treatment for the underlying cause (which they assumed to be present) was no acceptable treatment procedure and just caused unnecessary delay (Q09). Again, some respondents said they felt like part of an experiment in which other treatment options were tested out before effective treatment was provided (Q10).

Q08: For example, I tell them I have stomach aches or that something else hurts. Maybe there's a bacteria. However, they want to do some research [with different treatments] first. First they'll give you a painkiller to try, and if that doesn't work than we can try another one. If you've already had a light

For peer review only - http://bmjopen.bmj.com/site/about/guidelines.xhtml

medication, why don't they just try prescribing the other medicine that will have an effect?

[I-08, 2nd generation migrant, unemployed, difficulty with Dutch language]

Q09: They only thing they can tell you and do for you is give painkillers and paracetamol, nothing else. In the Netherlands, how can I say it, they only take care of your disease when it has progressed for quite a while.

[I-12, 1.5 generation migrant, unemployed, no difficulty with Dutch language]

Q10: I got a splint that would slip off after two steps. I went back and got another one. Exactly the same, after two steps it slipped off. I went back again. Listen, what are we up to here? What do you guys want? Am I the first person this happened to, that had to walk around with a splint? After great difficulty ... I got referred.... Listen, why didn't you guys give me that in the first place? Why? It was a mere €10 more.... They don't come through with the full treatment but do everything step by step. I don't mind that. I can understand that they don't

For peer review only - http://bmjopen.bmj.com/site/about/guidelines.xhtml

give my body an uppercut. I can understand that, but some things like those splints – why do you need to treat me like an experiment?

[I-05, 2nd generation migrant, paid work, no difficulty with Dutch language]

B. Healthcare utilisation in Turkey

Provoked by their experiences in the Dutch healthcare system (including perceived lack of recognition, mismatches in explanatory models of illness and the provision of unaccepted diagnoses), the majority of the respondents felt a strong need to consult the Turkish system, which they typically effectuated during an already scheduled holiday.

In Turkey, respondents as a rule felt that the care provided was more in line with the expectations they had of healthcare services, as they perceived fewer differences between their own explanatory model of illness and that of their Turkish physicians. That made them feel taken seriously, recognised as patients and treated as fully fledged individuals (Q11).

For peer review only - http://bmjopen.bmj.com/site/about/guidelines.xhtml

Q11: I notice when I'm in Turkey that they listen to me.... Okay, these are special clinics you visit, I understand that as well, but there they not only think about their own specialisation, but also outside of that.... It felt more like they took me seriously than they do here.

[I-03, 1rst generation migrant, paid work, no difficulty with Dutch language]

The respondents' narratives typically reflected a better match between their own explanatory models of illness and those of the Turkish healthcare system (Q12). Direct access to swift specialist diagnostics was seen as a way of shortcutting the stepwise diagnostics of Dutch general practitioners, in accordance with their felt urgency of risk reduction (Q13).

Q12: My [Dutch] GP, I told him something was wrong but I didn't know what. I told him it can't be good for your heart to beat at such a speed. He told me that's what it's made for. No, a heart is made to beat at a certain rhythm and to accelerate when you exercise or get frightened. It was made to function like that. I asked him what will happen if my heart goes on beating this fast? Yeah,

For peer review only - http://bmjopen.bmj.com/site/about/guidelines.xhtml

then maybe you'll live 50 years instead of 80. You can't give a patient an answer like that. To me that was the limit. So I took a plane to Istanbul and went to a cardiologist because I thought something was wrong with my heart. The doctor thought I'd already had some bloodwork done, but he didn't ask what the results were. I told him they couldn't find anything, but that I have a high heart rhythm and I'm tired. He did all the diagnostics a cardiologist would do: an ultrasound, an exercise stress test, a bicycle test, a lab workup. In the ultrasound they looked at the blood supply in my heart, looked for hardening of the arteries. Everything he told me put me at ease. He told me he could find nothing. He looked at my lab workup – he did a full workup – and he told me the problem was clear. He said they probably didn't test that. I had a vitamin D deficiency.

[I-03, 1rst generation migrant, paid work, no difficulty with Dutch language]

Q13: In the Netherlands they suspected my husband's pancreas, he was admitted to hospital, he had lost a lot of weight and they'd given him an appointment for a month later. He went to Turkey, got his results, went back

For peer review only - http://bmjopen.bmj.com/site/about/guidelines.xhtml

and gave them the report. The answer he got was that they [the Dutch hospital] were also planning to look at that. But it takes a whole month, maybe it could have metastasised by then?

[I-09, 2nd generation migrant, unemployed, no difficulty with Dutch language]

Almost all respondents reported that more effective curative treatments were provided in Turkey. They implied that they perceived provision of strong medication or an operation to be the only two decisive curative options for health improvement, thereby shortcutting the stepwise treatment approach of the Dutch healthcare system (Q14).

Q14: In Turkey they tell me I need an operation for my most recent symptoms. Even if they tell me no in the Netherlands, I'll go to Turkey for the necessary [operation]. Nothing more I can do.

[I-02, 1.5 generation migrant, unemployed, difficulty with the Dutch language]

For peer review only - http://bmjopen.bmj.com/site/about/guidelines.xhtml

All but one respondent thus saw treatment in Turkey as a valid option. This was limited perhaps by a lack of sufficient holidays and/or lack of a local social network for aftercare, but definitely not by financial restraints, even if specific services were not reimbursable (Q15).

> Q15: This year I was admitted to hospital [in Turkey] and they [Dutch insurance company] told me they might not pay for it. That doesn't interest me. Just let me know what's wrong and what we can do about it. I want to be free of the pain.... An MRI is just 400 lira, €50. I won't die from that expense, but getting an MRI in the Netherlands? ... I also bring back a lot of medicines from Turkey nowadays. [I-03, 1rst generation migrant, paid work, no difficulty with Dutch language]

For peer review only

C. Healthcare utilisation on return to the Netherlands

Upon their return to the Netherlands, almost all respondents went back to their Dutch GP to present Turkish test results, diagnoses and/or suggested treatments in order to discuss subsequent steps in the treatment process. This was not always without difficulty (Q16).

For peer review only - http://bmjopen.bmj.com/site/about/guidelines.xhtml

Q16: I had great difficulty convincing my GP that this was it. Couldn't be possible! I showed him the blood levels. I don't understand them, but according to my GP my vitamin B was above the Dutch recommended level. But the professor in Turkey had told me ... people from Turkey should have above 100.... I told my GP, listen, this and that is the case. She wouldn't believe it. But all right, it didn't matter, with great difficulty they gave me those 3 injections, once a week, and thank God, the symptoms were gone.

[I-05, 2nd generation migrant, paid work, no difficulty with Dutch language]

In case the consultation endpoint provided in Turkey was accepted by the Dutch healthcare provider, that justified the respondent's considerations for using Turkish health services and helped to compensate for perceived shortcomings in the Dutch system. At the same time, in case Turkish test results were not accepted, similar feelings of justification were expressed, as the fact that something had actually been found was perceived by respondents as comforting (Q17).

For peer review only - http://bmjopen.bmj.com/site/about/guidelines.xhtml

Q17: It's not nice to not be heard, that's what I notice in the Netherlands, be it a GP or a medical specialist. They're too simplistic about your issues, they send you away too easily and there's no alternative but to think up another solution.... Last time, my reassurance in Turkey was only because they do extensive testing. And even if it eventually turns out to be something small, they can explain my health issue with it. And if you treat that, it's over after that. That's my experience, that's why I prefer using healthcare in my country of origin. Nowadays I just buy a ticket without hesitating. My second opinion is now in Turkey.

[I-03, 1rst generation migrant, paid work, no difficulty with Dutch language]

Generally, respondents additionally argued that using healthcare in Turkey would be unnecessary and preventable if their GPs would just attend correctly to their health symptoms (Q18). Nonetheless, almost all respondents said they also appreciated the opportunity to discuss the test results and treatment options provided in Turkey upon their return in the Netherlands. As this was seen as a specific quality of Dutch general practitioners – being a case manager for their patients' health and illnesses –

For peer review only - http://bmjopen.bmj.com/site/about/guidelines.xhtml

the Turkish respondents continued to use primary as well as specialist healthcare in the Netherlands (Q19).

Q18: I had to go 3000 kilometres to learn that I have a vitamin D deficiency. No answer. ‘We didn’t think of that.’ I told them my body was giving a signal. They told me it was all in my mind. I got vitamin D drops. I noticed my heart rhythm was decreasing. So that was the cause of my problem. You’re inclined to start searching elsewhere. In the past 10 to 15 years I’ve been doing that more and more.

[I-03, 1rst generation migrant, paid work, no difficulty with Dutch language]

Q19: Also about my legs and sleeplessness. It had taken me years, and in Turkey the whole diagnosis took 2 hours and they told me I have restless legs. True, the whole night. He told me to take these pills, and thank God I still have those pills in case I need them. I went to the GP in the Netherlands and it all started up again from the beginning. Eventually she accepted it, but it had some side-effects. So again I’m happy with my GP, I can get along well with her, and

For peer review only - http://bmjopen.bmj.com/site/about/guidelines.xhtml

we're now trying the third medication because of the side-effects…. Now when I get side-effects, we try something else. That's also positive...

[I-05, 2nd generation migrant, paid work, no difficulty with Dutch language]

However, sometimes when the Turkish and Dutch systems provided conflicting diagnoses or treatment options, respondents were left in despair, unsure which version to believe. These few respondents nevertheless continued to see transnational healthcare use as one option to find solutions to their health problems (Q20).

Q20: If everything is fine, why do I have problems? I'm still suffering.... I'm at the point where I am losing myself. They couldn't find a solution to my headaches. For years, I've been going to doctors here as well as in Turkey. If need be I'll just have to go in Turkey as well.

[I-02, 1.5 generation migrant, not employed, difficulty with Dutch language]

For peer review only - http://bmjopen.bmj.com/site/about/guidelines.xhtml

DISCUSSION

Summery of the findings

In the stories of Dutch residents with a Turkish ethnic background, this study observed how: (A) cross-border healthcare use was encouraged by cultural mismatches between expected and provided services and by differing explanatory models of illness upheld by patients and Dutch providers; (B) both transnationalism in patients and entitlements to insurance reimbursement facilitated the use of Turkish health services to bypass perceived barriers in the Dutch system; (C) cultural mismatches were reinforced during GP consultations after the patients' return to the Netherlands, thereby inducing further service use abroad.

Interpretation

Our findings indicate that the regular transnational healthcare use of Dutch residents with a Turkish ethnic background (15) may be the result of mismatches between the patients' and physicians' explanatory models of illness in relation to cultural differences in both healthcare systems and national cultures. First, the feeling of our respondents that their illness sensations were not easily acknowledged as symptoms

For peer review only - http://bmjopen.bmj.com/site/about/guidelines.xhtml

reflects that the interpretation of such sensations may heavily depend on culture (22). Our findings and previous studies suggest that mismatches in explanatory models may result from a perceived cultural distance to the Dutch healthcare system (16) rather than from a perceived cultural distance to the Dutch society (30). Second, such mismatches made some of our respondents feel a second-class citizen. In this respect, another Dutch qualitative study found that Dutch patients from Turkish origin regularly reported that they were being treated indifferently, inattentively or discriminatorily by physicians in primary care, while native Dutch patients did not mention such experiences (31).

Despite the mismatches in explanatory models of illness, most of the respondents in our study continued to see their GP as their case manager and a reliable source in seeking care. This means that the GP in the Netherlands, and perhaps also in other counties with a similar gatekeeper system, could play key role in reducing transnational healthcare use. Although cultural gaps may be difficult to bridge (26), cross-border healthcare utilization also rests on individual beliefs, motivation and decisions (20, 21). Therefore, one approach could be to strengthen patient centred primary care (32), by providing culturally sensitive and competent

For peer review only - http://bmjopen.bmj.com/site/about/guidelines.xhtml

For peer review only

care during the consultation, as to achieve mutual understanding between the patient and the GP (22, 33). First, this includes being aware of cultural differences, such as in uncertainty avoidance (28), and the related need to find pathophysiological causes of sensations and symptoms. Second, it requires cross-cultural communication (33), such as discussing mismatches and negotiating the need for transnational healthcare use (22), including possible iatrogenic outcomes and timely seeking professional help if these might occur (5).

However, providing culturally sensitive care alone may not be sufficient to change the transnational healthcare use of Dutch residents of Turkish origin. Our results confirm that the insurers' reimbursement of healthcare and/or the low cost of care in the country of origin may play an equally important part in transnational healthcare uptake (1, 34). These instruments made opting for health services in Turkey feel natural to our respondents. For Dutch insurance companies, one option to limit the higher than average healthcare use could be contracting only a selection of care providers for the delivery of care to people with Dutch insurance (13). Currently, insurance companies reimburse services from all possible providers abroad, which can be justified by comparable scores on quality outcome indicators

For peer review only - http://bmjopen.bmj.com/site/about/guidelines.xhtml

(25), while in the Netherlands they only reimburse care from “preferred providers” meeting additional quality process standards (26). Introducing similar quality procedures for the delivery of healthcare in countries abroad could also contribute to a further harmonisation between services in different countries as well as to cost-effective care, while the patient’s option to obtain care abroad is being maintained. To additionally minimise the risks of medicalisation and adverse iatrogenic effects, and to coordinate transnational diagnostics and treatment, an online portal for international medical information transfer could be considered, although achieving personal data safety in conformity with privacy legislations may be challenging.

Strength and limitations

Our main strength is the use of a cultural systems perspective (22, 35). This helped us to see how transnational healthcare use may be encouraged by an interaction of factors in both clinical practice, healthcare system and national culture. Another strength lies in the ethnic-concordant interviewer (AȘ), who was familiar with respondents’ language and cultural expressions. As the biographic-narrative interpretive method (29, 36, 37) led to in-depth and meaningful information, we feel

For peer review only - http://bmjopen.bmj.com/site/about/guidelines.xhtml

confident to have successfully addressed the potential downsides of such an interviewer, by both reassuring anonymity and letting respondents have ownership of the interviews.

An important limitation is the rather small number of participants in the study, sampled from two primary care practices, in one transnational healthcare context. This limits the possibility to generalize our findings to other Dutch primary care patients of Turkish background as well as to other patient populations, both within and beyond our country. Although the pattern in transnational healthcare use we found was pronounced, additional research in other populations and transnational healthcare contexts seems warranted.

A second limitation is that we only included patients with a Turkish background and no patients of Dutch ethnic origin. Therefore, we cannot say that it is especially Dutch patients of Turkish origin that bypass the general practitioner to directly access specialist care. Similarly, we cannot tell whether the Dutch patients of Turkish origin, while visiting Turkish health care services, were treated differently from the Turkish residents without a migration history. As patient satisfaction can increase when such

For peer review only - http://bmjopen.bmj.com/site/about/guidelines.xhtml

a bypass option is available (38), it would be interesting to compare our findings with data from patients of Dutch ethnic origin.

Conclusions

Our study uncovered how transnational healthcare use of Dutch patients with a Turkish background was encouraged by mismatches with their Dutch general practitioner. Their dissimilar explanatory models of illness reflected differences in national healthcare systems and national cultures. In order to reduce the unwelcome consequences of transnational healthcare use, measures may include strengthening the provision of culturally sensitive care in the country of residence, and restricting the reimbursement of care in the country origin while maintaining the option to obtain care abroad. Future research could include other migrant populations, qualitatively compare the healthcare experiences of migrant and native communities, and quantitatively assess the importance of the patterns we identified.

For peer review only - http://bmjopen.bmj.com/site/about/guidelines.xhtml

Acknowledgements

We are most grateful for the investments of time and energy made by the respondents in sharing their stories and welcoming the first author into their homes. Without them, this study would not have been possible.

Funding

The first author was a PhD candidate at the Amsterdam UMC, University of Amsterdam, with research funded by a MD-PhD fellowship from the same institution. Grant number: N/A.

Competing interest statement

None declared.

Availability of data and materials

The interview transcripts and the data analysis in the MaxQDA project file are not publicly available. In order to build the required trust, the respondents' informed

For peer review only - http://bmjopen.bmj.com/site/about/guidelines.xhtml

consent remained restricted to the use of the interview data for the present study only.

Authors contributions

AȘ collected the data, analysed the interviews and drafted the manuscript. JH read the coded interviews and reflected on the analysis and interpretation of the data. KS and RJGP contributed to the conception and design of the study. KS also provided the daily supervision of the fieldwork. All authors were involved in revising initial drafts of the manuscript, and read and approved the final manuscript.

Ethics statement

According to the Dutch Medical Research Involving Human Subjects Act, this interview study did not require approval by a medical research ethics committee. Data and analysis logs were stored in a protected digital environment, in accordance with Amsterdam University Medical Centers guidelines and Dutch and EU privacy legislation.

For peer review only - http://bmjopen.bmj.com/site/about/guidelines.xhtml

REFERENCES

1. Villa-Torres L, González-Vázquez T, Fleming PJ, González-González EL, Infante-Xibille C, Chavez R, et al. Transnationalism and health: A systematic literature review on the use of transnationalism in the study of the health practices and behaviors of migrants. Soc Sci Med. 2017;183:70-9.

2. Portes A. Globalization from below. The rise of transnational communities. Princeton: Princeton University; 1997.

3. Lee JY, Kearns RA, Friesen W. Seeking affective health care: Korean immigrants' use of homeland medical services. Health Place. 2010;16(1):108-15.

4. Busfield J. The concept of medicalisation reassessed. Sociol Health Illn. 2017;39(5):759-74.

5. Moynihan R, G⦰tzsche PC, Heath I, Henry D. Selling sickness: the pharmaceutical industry and disease mongeringCommentary: Medicalisation of risk factors. BMJ. 2002;324(7342):886.

6. Foley BM, Haglin JM, Tanzer JR, Eltorai AEM. Patient care without borders: a systematic review of medical and surgical tourism. J Travel Med. 2019;26(6).

For peer review only - http://bmjopen.bmj.com/site/about/guidelines.xhtml

7. Şekercan A, Woudstra AJ, Peters RJG, Lamkaddem M, Akgün S, Essink-Bot ML. Dutch citizens of Turkish origin who utilize healthcare services in Turkey: a qualitative study on motives and contextual factors. BMC Health Serv Res. 2018;18(1):289.

8. Xu T, Wang W, Du J. An Integrative Review of Patients' Experience in the Medical Tourism. Inquiry. 2020;57:46958020926762-.

9. Hanefeld J, Smith R, Horsfall D, Lunt N. What Do We Know About Medical Tourism? A Review of the Literature With Discussion of Its Implications for the UK National Health Service as an Example of a Public Health Care System. Journal of Travel Medicine. 2014;21(6):410-7.

10. Statistics Netherlands. File Asylum, migration and integration 2021 [Available from: https://www.cbs.nl/nl-nl/dossier/dossier-asiel-migratie-en-integratie/.

11. Stronks K, Snijder MB, Peters RJ, Prins M, Schene AH, Zwinderman AH. Unravelling the impact of ethnicity on health in Europe: the HELIUS study. BMC Public Health. 2013;13:402.

12. European Union. European Health Insurance Card 2019 [Available from: https://ec.europa.eu/social/main.jsp?catId=559.

For peer review only - http://bmjopen.bmj.com/site/about/guidelines.xhtml

13. Healthcare Insurance Company CZ. Healthcare Insurance Abroad 2019 [Available from: https://www.cz.nl/zorgverzekering/buitenland.

14. Eurostat. Migrants in Europe. A statistical portrait of the first and second generation. 2011.

15. Șekercan A, Snijder MB, Peters RJ, Stronks K. Associations between healthcare consumption in country of origin and in country of residence by people with Turkish and Moroccan backgrounds living in the Netherlands: the HELIUS study. Eur J Public Health. 2019;29(4):694-9.

16. Șekercan A, Lamkaddem M, Snijder MB, Peters RJ, Essink-Bot ML. Healthcare consumption by ethnic minority people in their country of origin. Eur J Public Health. 2015;25(3):384-90.

17. Smits FT, Brouwer HJ, Zwinderman AH, Mohrs J, Smeets HM, Bosmans JE, et al. Morbidity and doctor characteristics only partly explain the substantial healthcare expenditures of frequent attenders: a record linkage study between patient data and reimbursements data. BMC Family Practice. 2013;14(1):138.

For peer review only

18. Wang L, Kwak MJ. Immigration, barriers to healthcare and transnational ties: A case study of South Korean immigrants in Toronto, Canada. Soc Sci Med. 2015;133:340-8.

19. Lokdam N, Kristiansen M, Handlos LN, Norredam M. Use of healthcare services in the region of origin among patients with an immigrant background in Denmark: a qualitative study of the motives. BMC Health Services Research. 2016;16(1):99.

20. Norris P, Fa'alau F, Va'ai C, Churchward M, Arroll B. Navigating Between Illness Paradigms: Treatment Seeking by Samoan People in Samoa and New Zealand. Qualitative Health Research. 2009;19(10):1466-75.

21. Weerasinghe MC, Fernando DN. Paradox in treatment seeking: an experience from rural Sri Lanka. Qual Health Res. 2011;21(3):365-72.

22. Kleinman A. Concepts and a model for the comparison of medical systems as cultural systems. Social Science & Medicine Part B: Medical Anthropology. 1978;12:85-93.

23. Kleinman A, Eisenberg L, Good B. Culture, illness, and care: clinical lessons from anthropologic and cross-cultural research. Ann Intern Med. 1978;88(2):251-8.

For peer review only - http://bmjopen.bmj.com/site/about/guidelines.xhtml

24. Hay MC. Reading Sensations: Understanding the Process of Distinguishing `Fine' from `Sick'. Transcultural Psychiatry. 2008;45(2):198-229.

25. Joint Commision International. JCI-Accredited Organizations 2019 [Available from: https://www.jointcommissioninternational.org/about-jci/accredited-organizations/.

26. Kroneman M, Boerma W, Berg Mvd, Groenewegen P, Jong Jd, Ginneken Ev. The Netherlands: health system review. Health system in transition. Kopenhagen: WHO Regional Office for Europe; 2016.

27. OECD & European Union. Health at a glance: Europe 2017 2017 [Available from: https://www.oecd-ilibrary.org/social-issues-migration-health/health-at-a-glance-2017_health_glance-2017-en.

28. Hofstede G. Culture's consequences: Comparing values, behaviors, institutions and organizations across nations. Thousand Oaks, CA: Sage; 2001.

29. Wengraf T. Qualitative research interviewing: Biographic narrative and semistructured methods. Thousand Oaks, CA: Sage; 2001.

30. Fassaert T, De Wit MA, Tuinebreijer WC, Knipscheer JW, Verhoeff AP, Beekman AT, et al. Acculturation and psychological distress among non-Western

For peer review only

For peer review only - http://bmjopen.bmj.com/site/about/guidelines.xhtml

Muslim migrants--a population-based survey. Int J Soc Psychiatry. 2011;57(2):132-43.

31. Schinkel S, Schouten BC, Kerpiclik F, Van Den Putte B, Van Weert JCM. Perceptions of Barriers to Patient Participation: Are They Due to Language, Culture, or Discrimination? Health Commun. 2019;34(12):1469-81.

32. Davis K, Schoenbaum SC, Audet AM. A 2020 vision of patient-centered primary care. J Gen Intern Med. 2005;20(10):953-7.

33. Watt K, Abbott P, Reath J. Developing cultural competence in general practitioners: an integrative review of the literature. BMC Family Practice. 2016;17(1):158.

34. Su D, Richardson C, Wen M, Pagán JA. Cross-border utilization of health care: evidence from a population-based study in south Texas. Health Serv Res. 2011;46(3):859-76.

35. Carey JW. Linking Qualitative and Quantitative Methods: Integrating Cultural Factors into Public Health. Qualitative Health Research. 1993;3(3):298-318.

36. Morse JM. The Cultural Sensitivity of Grounded Theory. Qualitative Health Research. 2001;11(6):721-2.

For peer review only

For peer review only - http://bmjopen.bmj.com/site/about/guidelines.xhtml

37. Berger R. Now I see it, now I don't: researcher's position and reflexivity in qualitative research. Qualitative Research. 2015;15(2):219-34.

38. Aoki T, Yamamoto Y, Ikenoue T, Kaneko M, Kise M, Fujinuma Y, et al. Effect of Patient Experience on Bypassing a Primary Care Gatekeeper: a Multicenter Prospective Cohort Study in Japan. J Gen Intern Med. 2018;33(5):722-8.

For peer review only

For peer review only - http://bmjopen.bmj.com/site/about/guidelines.xhtml

Table 1. Characteristics of interviewed respondents

	Summarised characteristics		
Participants (number)	13		
Age (range)	39–78		
Gender (% female)	62%		
Migration generation[1] (% 1st, 1.5, 2nd)	31	23	46
Employment status (% paid, % unemployed, % retired)	38	54	8
Educational attainment level[2] (% low, middle, high)	70	30	0
Number of years in Netherlands (% born in NL, range)	31	24–54	
Number of yearly visits to Turkey (range)	0.5–4		
Preference to stay in which country[3] (% NL, % TR, % circular)	23	23	54
Number of diseases (range)	0–6		
Difficulty with Dutch language (% yes)	31		

[1] 1.5 migration generation: migration to Netherlands before age 12

For peer review only - http://bmjopen.bmj.com/site/about/guidelines.xhtml

[2] Low: primary or less education; middle: lower general or vocational secondary; high: upper general or vocational secondary or tertiary

[3] Circular: preference to stay longer periods of time in both countries

For peer review only

For peer review only - http://bmjopen.bmj.com/site/about/guidelines.xhtml

Supplementary Material I. Interview approach

We used the biographic-narrative interview method (1). The first part of the interview was an open interview, the second part was structured around the episodes of health care use that the respondent had mentioned in the first part.

Part 1. Open query
"Lütfen bana hayatınızın hikayesini, sizin için sağlık, hastalık ve bakım ilgili önemli olan tüm olayları ve yasadıklarınızı bana anlatın. Başlamak istediğiniz yerde başlayın, ve istediniz kadar anlatın, vaktim çok, ben siz anlatınız an, sissiz kalıyorum, sadece sonra için birkaç not alacağım"

"Please tell me the story of your life, in the sense of all the events and experiences that have been important to you personally regarding health, sickness and seeking care. Start wherever you want to, and tell me as much as you want, I have a lot of time, and if I get confused, I'll make some notes for later"

Part 2. In-depth questions
Questions to further explore the respondent's experiences during the subsequent episodes of healthcare use. These questions were not pre-determined, but guided by (a) the topics raised by the respondent; and (b) the exact wording used by the respondent. In order to understand the respondent's experiences and explanatory model of illness, this part of the interview was structured around the three phases of the consultation (2, 3): (I) the presentation of illness sensations; (II) the transition of an illness into a disease; (III) presenting a treatment regime.

1. Wengraf T. Qualitative research interviewing: Biographic narrative and semistructured methods. Thousand Oaks, CA: Sage; 2001.
2. Kleinman A. Concepts and a model for the comparison of medical systems as cultural systems. Social Science & Medicine Part B: Medical Anthropology. 1978;12:85-93.
3. Hay MC. Reading Sensations: Understanding the Process of Distinguishing `Fine' from `Sick'. Transcultural Psychiatry. 2008;45(2):198-229.

For peer review only - http://bmjopen.bmj.com/site/about/guidelines.xhtml

Supplementary Material II. Codes used in the Qualitative Analysis

Steps of consultation (negotiation at each phase)	
	Illness presentation and persuading
	illness to disease translation
	Acceptable treatment regimen
Sensation to symptom	
	Vulnerability
	Urgency
	Disability/death (seriousness)
Core adaptive tasks of healthcare systems	
	Dealing with illness
	Healthcare strategies
	Consultation
	Healing activities
	Health influencing behaviours
	Dealing with healthcare outcomes
Explanatory model	
	Patient
	Provider
	Differences/troubles
	Agreement
Endpoint	
	Conflicting
	Unacceptable
	Acceptable
	Test result
	Diagnosis
	It's nothing serious
Turkish Healthcare	
	Direct access to specialist care
	Ruling in versus ruling out
	Interventionalist
	Value for money
	"Broad" & fast diagnostics
Dutch Healthcare	
	Specialist referral
	Ruling out versus ruling in
	Non-invertentionalist
	Stepwise diagnosis
	Stepwise treatment
Life World Intern	
	Turkish examples
	Dutch examples
	Cultural assumptions
Life World Extern	

For peer review only - http://bmjopen.bmj.com/site/about/guidelines.xhtml

Dutch setting experience
Turkish setting experience
Cultural context
Being an experiment
Self-reliance
Finances
Language
Delay
Shopping (behaviour)
Uncertainty avoidance
Transnationalism
Access without barriers<br>Why? Expressing lack of understanding<br>Metaphor

For peer review only - http://bmjopen.bmj.com/site/about/guidelines.xhtml

Consolidated criteria for reporting qualitative studies (COREQ): 32-item checklist

Developed from: Tong A, Sainsbury P, Craig J. Consolidated criteria for reporting qualitative research (COREQ): a 32-item checklist for interviews and focus groups. *International Journal for Quality in Health Care*. 2007. Volume 19, Number 6: pp. 349 – 357

YOU MUST PROVIDE A RESPONSE FOR ALL ITEMS. ENTER N/A IF NOT APPLICABLE

No. Item	Guide questions/description	Reported on Page #
Domain 1: Research team and reflexivity		
Personal Characteristics		
1. Inter viewer/facilitator	Which author/s conducted the interview or focus group?	9
2. Credentials	What were the researcher's credentials? E.g. PhD, MD	1
3. Occupation	What was their occupation at the time of the study?	10-11
4. Gender	Was the researcher male or female?	10-11
5. Experience and training	What experience or training did the researcher have?	10-11
Relationship with participants		
6. Relationship established	Was a relationship established prior to study commencement?	8, 10-11
7. Participant knowledge of the interviewer	What did the participants know about the researcher? e.g. personal goals, reasons for doing the research	10-11
8. Interviewer characteristics	What characteristics were reported about the inter viewer/facilitator? e.g. Bias, assumptions, reasons and interests in the research topic	10-11
Domain 2: study design		
Theoretical framework		
9. Methodological orientation and Theory	What methodological orientation was stated to underpin the study? e.g. grounded theory, discourse analysis, ethnography, phenomenology, content analysis	6-8, 9
Participant selection		
10. Sampling	How were participants selected? e.g. purposive, convenience, consecutive, snowball	8
11. Method of approach	How were participants approached? e.g. face-to-face, telephone, mail, email	8
12. Sample size	How many participants were in the study?	8
13. Non-participation	How many people refused to participate or dropped out? Reasons?	8
Setting		
14. Setting of data collection	Where was the data collected? e.g. home, clinic, workplace	8
15. Presence of non-participants	Was anyone else present besides the participants and researchers?	8-9
16. Description of sample	What are the important characteristics of the sample? e.g. demographic data, date	11, 25

Data collection		
17. Interview guide	Were questions, prompts, guides provided by the authors? Was it pilot tested?	9
18. Repeat interviews	Were repeat interviews carried out? If yes, how many?	N/A
19. Audio/visual recording	Did the research use audio or visual recording to collect the data?	8
20. Field notes	Were field notes made during and/or after the interview or focus group?	N/A
21. Duration	What was the duration of the interviews or focus group?	8
22. Data saturation	Was data saturation discussed?	10
23. Transcripts returned	Were transcripts returned to participants for comment and/or correction?	N/A
Domain 3: analysis and findings		
Data analysis		
24. Number of data coders	How many data coders coded the data?	9-10
25. Description of the coding tree	Did authors provide a description of the coding tree?	9-10; Supplementary Material I
26. Derivation of themes	Were themes identified in advance or derived from the data?	9-10
27. Software	What software, if applicable, was used to manage the data?	9-10
28. Participant checking	Did participants provide feedback on the findings?	N/A
Reporting		
29. Quotations presented	Were participant quotations presented to illustrate the themes/findings? Was each quotation identified? e.g. participant number	26-33
30. Data and findings consistent	Was there consistency between the data presented and the findings?	11-15, 26-33
31. Clarity of major themes	Were major themes clearly presented in the findings?	11-15
32. Clarity of minor themes	Is there a description of diverse cases or discussion of minor themes?	11-15

Once you have completed this checklist, please save a copy and upload it as part of your submission. When requested to do so as part of the upload process, please select the file type: *Checklist*. You will NOT be able to proceed with submission unless the checklist has been uploaded. Please DO NOT include this checklist as part of the main manuscript document. It must be uploaded as a separate file.

For peer review only

For peer review only - http://bmjopen.bmj.com/site/about/guidelines.xhtml
